# Somatotopic disruption of the functional connectivity of the primary sensorimotor cortex in complex regional pain syndrome type 1

**DOI:** 10.1002/hbm.26513

**Published:** 2023-10-14

**Authors:** Jaakko Hotta, Jukka Saari, Hanna Harno, Eija Kalso, Nina Forss, Riitta Hari

**Affiliations:** ^1^ Department of Neuroscience and Biomedical Engineering Aalto University School of Science Espoo Finland; ^2^ Aalto NeuroImaging Aalto University Espoo Finland; ^3^ Department of Neurology Helsinki University Hospital and Clinical Neurosciences, Neurology, University of Helsinki Helsinki Finland; ^4^ Department of Applied Physics University of Eastern Finland Kuopio Finland; ^5^ Department of Anaesthesiology, Intensive Care and Pain Medicine University of Helsinki and Helsinki University Hospital Helsinki Finland; ^6^ Department of Art and Media Aalto University School of Arts, Design and Architecture Helsinki Finland

**Keywords:** chronic pain, complex regional pain syndrome, CRPS, fMRI, functional connectivity, primary sensorimotor cortex, sensorimotor network

## Abstract

In complex regional pain syndrome (CRPS), the representation area of the affected limb in the primary sensorimotor cortex (SM1) reacts abnormally during sensory stimulation and motor actions. We recorded 3T functional magnetic resonance imaging resting‐state data from 17 upper‐limb CRPS type 1 patients and 19 healthy control subjects to identify alterations of patients' SM1 function during spontaneous pain and to find out how the spatial distribution of these alterations were related to peripheral symptoms. Seed‐based correlations and independent component analyses indicated that patients' upper‐limb SM1 representation areas display (i) reduced interhemispheric connectivity, associated with the combined effect of intensity and spatial extent of limb pain, (ii) increased connectivity with the right anterior insula that positively correlated with the duration of CRPS, (iii) increased connectivity with periaqueductal gray matter, and (iv) disengagement from the other parts of the SM1 network. These findings, now reported for the first time in CRPS, parallel the alterations found in patients suffering from other chronic pain conditions or from limb denervation; they also agree with findings in healthy persons who are exposed to experimental pain or have used their limbs asymmetrically. Our results suggest that CRPS is associated with a sustained and somatotopically specific alteration of SM1 function, that has correspondence to the spatial distribution of the peripheral manifestations and to the duration of the syndrome.

## Graphical abstract

This study demonstrates disturbances in the function and functional connectivity of the primary sensorimotor cortex of complex regional pain syndrome patients, likely contributing to their spontaneous pain.

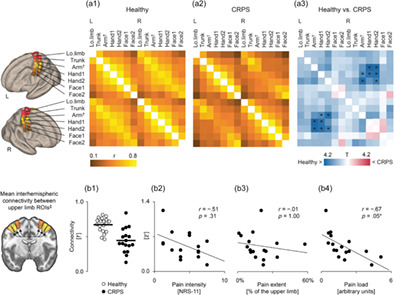

## 1 INTRODUCTION

Complex regional pain syndrome (CRPS) is characterized by limb‐pain that is accompanied with varying degrees of other sensory, motor, and autonomic symptoms. The pain is typically enhanced by limb movements and sensory stimuli (de Boer et al., 2011; Hotta et al., 2015; Veldman et al., 1993) but is also present spontaneously during rest. Although the pathophysiology of CRPS is still incompletely understood, alterations of the central nervous system are thought to be instrumental in maintaining and modulating the syndrome. In particular, a role of the primary sensorimotor cortex (SM1) in pain and other symptoms has been studied extensively during limb movements and sensory stimulation (e.g., Hotta et al., 2016; Juottonen et al., 2002; Maihöfner et al., 2005). Less is known of the SM1 alterations during spontaneous unprovoked pain.

Resting‐state functional magnetic resonance imaging (fMRI) of healthy individuals has unraveled several brain‐networks that are synchronized at low frequencies (<0.1 Hz) as a sign of “functional connectivity” (FC). One example is the sensorimotor network (SMN) that involves the SM1 of both hemispheres (Beckmann et al., 2005), with prominent interhemispheric connectivity between homotopic areas (Stark et al., 2008; van den Heuvel & Pol, 2010). In healthy individuals, this connectivity is robust and stable at rest, but modulated somatotopically by motor actions and somatosensory stimulation. For example, disuse of one hand (by applying a unilateral cast) associated with compensatory overuse of the other hand, hampers the interhemispheric FC between SM1 hand representation areas (Newbold et al., 2020). In turn, experimentally induced sustained pain disengages the SM1 representation area of the painfully stimulated limb from the rest of the SMN and instead increases its FC with the right anterior insula (aINS), a node of the salience network (Kim et al., 2013). Given that continuous limb disuse and pain characterize CRPS, patients' SMN connectivity could be somatotopically disrupted in correspondence with the peripheral symptoms. Evidence of such a disruption would provide valuable insight into the role of the SM1 in CRPS but earlier studies have not specifically addressed somatotopy of the connectivity alterations in CRPS (Azqueta‐Gavaldon et al., 2019; Baliki et al., 2014; Becerra et al., 2014; Bolwerk et al., 2013; Kim et al., 2018; Shokouhi et al., 2018; van Velzen et al., 2016).

In the current study, we therefore recorded resting‐state fMRI from patients with upper‐limb CRPS to explore alterations in their SM1 connectivity. We specifically tested the hypothesis that the somatotopic distribution of cortical alterations is associated with the peripheral symptoms. We searched for FC alterations (1) between SM1 representation areas of different body parts, (2) between SM1 and insula, and (3) between SM1 upper‐limb areas and all other parts of the brain, as well as (4) of the SMN in general. We further examined the covariation of the observed FC alterations with pain intensity, spatial extent of pain, motor symptoms, and disease duration.

## 2 MATERIALS AND METHODS

### 2.1 Subjects

**FIGURE 1:**
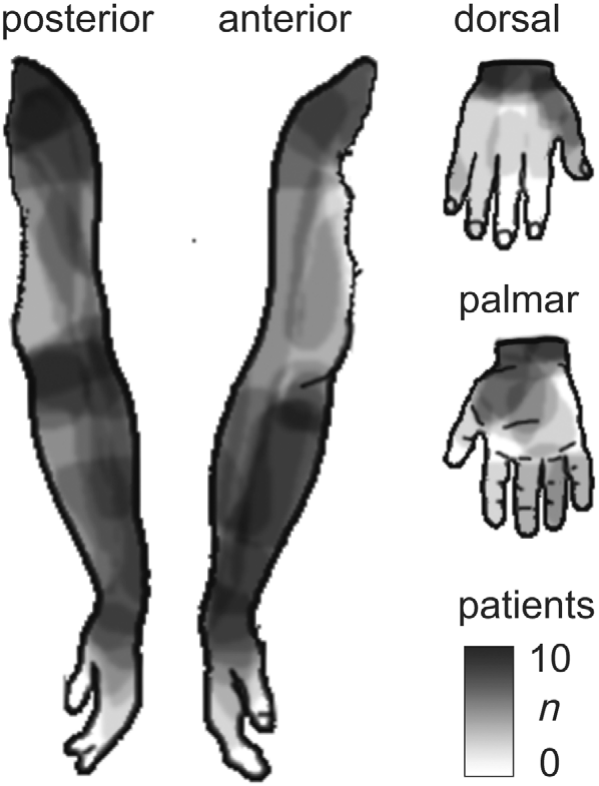
The distribution of pain in the upper limb of the complex regional pain syndrome patient group (*n* = 17). For visualization, the pain distribution of the patients with left‐sided symptoms has been mirrored to the right upper‐limb template. The black–white gradient indicates the number of patients reporting pain in each area. The distributions are based on the patients' free‐hand drawings of experienced pain (1) on a template of the whole human body and (2) on more detailed templates of both hands.

We studied 17 unilateral upper‐limb CRPS type 1 patients (mean ± SD age 44 ± 9 years, median 45, range 24–58; 1 male) and 19 healthy control subjects (mean ± SD age 44 ± 9 years, median 46, range 25–60; 1 male); ages did not differ between the groups (*t* = 0.36, *p* = .92, two‐tailed independent sample *t*‐test). All subjects were right‐handed according to the Edinburgh Handedness Inventory. Twelve patients (71%) had CRPS in their right upper limb and five (29%) in the left one. Table 1 presents the clinical data of the patients and Figure 1 summarizes the distribution of pain in the upper limb.

**TABLE 1:** Clinical data of the patients.

Patient	Age (years)	Duration of CRPS (years)	Dominant hand	Side of CRPS	CRPS symptoms/signs				Spontaneous pain before fMRI (NRS 0–10)
					Sensory	Motor or trophic	Vasomotor	Edema or sudomotor	
p01	46.0	1.4	R	R	+/+	+/+	+/+	+/−	3
p02	38.1	1.5	R	R	+/+	+/+	+/−	+/−	6
p03	48.1	2.0	R	R	+/+	+/+	+/+	+/−	4
p04	50.4	2.1	R	R	+/+	+/+	+/+	+/+	5
p05	47.8	3.2	R	R	+/+	+/+	+/+	+/−	7
p06	35.9	3.3	R	R	+/+	+/+	+/+	+/+	7
p07	57.9	5.0	R	R	+/+	+/+	+/−	−/−	7
p08	44.5	7.5	R	R	+/+	+/−	+/+	+/+	5
p09	43.1	8.2	R	R	+/+	+/+	+/+	+/−	5
p10	44.1	8.3	R	R	+/+	+/+	+/+	+/−	7
p11	56.6	11.8	R	R	+/+	+/+	+/+	+/+	7
p12	47.1	15.5	R	R	+/+	+/−	+/+	+/−	3
p13	24.2	1.5	R	L	+/+	+/+	+/+	+/−	3
p14	31.0	3.5	R	L	+/+	+/+	+/+	+/+	8
p15	49.5	3.5	R	L	+/+	+/+	+/+	+/+	6
p16	44.7	4.2	R	L	+/+	+/+	+/+	+/+	8
p17	34.9	4.5	R	L	+/+	+/+	+/+	+/−	4

*Note*: These data have been presented in studies published earlier on the same patient cohort (Hotta et al., 2015, 2016, 2017; Zhou et al., 2015).

Abbreviations: −/−, neither symptoms nor signs; +/−, symptoms; +/+, symptoms and signs; CRPS, complex regional pain syndrome; fMRI, functional magnetic resonance imaging; L, left; NRS, numeric rating scale; R, right.

Inclusion criteria for patients were (1) the diagnosis of upper‐limb CRPS type 1, (2) moderate or severe pain at rest or movement (≥5 on an 11‐point numerical rating scale [NRS]; 0 = no pain, 10 = extreme pain), (3) duration of CRPS of >6 months, (4) right‐handedness, (5) age 18–65 years, (6) no other major neurological or psychiatric diagnosis, (7) no drug or alcohol addiction, and (8) no contraindication for MRI. From the pool of 96 CRPS patients treated at the Pain Clinic of the Helsinki University Hospital, and from 19 patients referred to us from other clinics in the Helsinki and Uusimaa district, we invited 35 eligible candidates for examination. From these, 17 patients who fulfilled the inclusion criteria and were willing to participate were included in the study.

At the inclusion examination, the patients were interviewed and clinically examined by a neurologist. Sixteen of the 17 patients fulfilled the “research criteria” and one the less stringent “clinical criteria” of CRPS (Harden et al., 2007). Electromyoneurography did not show signs of nerve injury, and all patients were classified as having CRPS type 1.

All patients used regular and/or on‐demand medication to treat their pain: mild opioids (10 patients), gabapentinoids (8), nonsteroidal anti‐inflammatory drugs (7), acetaminophen (6), tricyclic antidepressants (5), serotonin‐norepinephrine reuptake inhibitors (3), tizanidine (2), buprenorphine (2), mirtazapine (1), and lamotrigine (1).

To assess motor symptoms, patients completed an upper‐limb‐disability questionnaire (Disabilities of Arm, Shoulder, and Hand [DASH]: Institute for Work & Health http://www.dash.iwh.on.ca/home; Hudak et al., 1996) and rated their maximum pain intensity when moving the affected limb during the previous week on an NRS. A physiotherapist assessed the motor function of the affected limb by measuring hand dexterity with the nine‐hole peg test (Mathiowetz et al., 1985), hand grip‐strength with Jamar dynamometer (position II), and active range of motion (AROM) of the wrist. From these results, we calculated a motor symptom severity index (MSSI) by summing up the z‐scores of the questionnaires and motor measures for each patient (for details, see Hotta et al., 2017). For the sum, the z‐scores of the AROM and grip strength measures were multiplied by −1, so that a higher MSSI corresponded with more severe motor symptoms.

This study was approved by the Ethics Committee, Department of Medicine of the Helsinki and Uusimaa Hospital District and was conducted according to the Declaration of Helsinki. Prior to participation, subjects gave written informed consent. Subjects were recruited and the data were collected between January 1, 2011, and January 30, 2013.

### 2.2 Experiment

Prior to the fMRI measurements, the patients reported their pain intensity at rest on a NRS (0–10; Table 1).

The fMRI acquisition took place at the Advanced Magnetic Imaging Centre of Aalto NeuroImaging, Aalto University. Due to scanner upgrade during our study, 12 (71%) patients and 14 (74%) healthy control subjects were measured with a Signa HDxt 3.0T scanner (GE Healthcare, Milwaukee, Wisconsin) with a 16‐channel head coil, and five patients (29%) and five healthy control subjects (26%) with a Magnetom Skyra 3T scanner (Siemens Healthcare, Erlangen, Germany) with a 30‐channel head coil (modified from the 32‐channel coil to optimize field of vision).

The following parameters were applied for functional T2*‐weighted gradient‐echo echo‐planar images: TR 2.5 s, TE 30 ms, flip angle 75°, matrix size 64 × 64, field of view 24 cm, slice thickness 3.0 mm, in‐plane resolution 3.75 × 3.75 mm^2^ with no gap, number of slices 50 (GE) or 47 (Siemens). The number of time points was 190. The first four images were discarded to ensure stabilization of the MR signal. During the same scanning session, anatomical high‐resolution 1 × 1 × 1 mm^3^ T1‐weighted MR images (176 slices with matrix size of 256 × 256) were acquired, using ultrafast gradient‐echo 3D sequences (3D fast SPGR with GE scanner, MPRAGE with Siemens scanner) with TR 10.0/2530 ms, TE 3.0/3.3 ms, flip angle 15°/7° for GE/Siemens.

During the 8‐min resting‐state fMRI measurement, the subjects were instructed to avoid head and hand movements, to remain awake and to keep eyes open and fixated on a cross projected on a screen 34 cm in front of their eyes. Soft padding inside the head coil minimized involuntary head movements. We monitored signs of drowsiness (transient eye closure) with an infrared eye camera (iView X MRI‐LR, SensoMotoric Instruments GmbH, Germany in GE scanner, and EyeLink 1000, SR Research Ltd., Ontario, Canada in Siemens scanner). We also monitored hand movements by fixing MR‐compatible custom‐made accelerometers to both hands (Velcro strap around fingers 3–5) and leading the signals to BrainAmp ExG MR amplifiers (Brain Products GmbH, Munich, Germany).

### 2.3 MRI analysis

#### 2.3.1 Preprocessing

We preprocessed the fMRI data with SPM8 (http://www.fil.ion.ucl.ac.uk/spm/software/spm8/), including slice‐time correction, motion correction (realignment), and coregistration of the mean functional images with skull‐stripped T1 images. The T1 images were segmented into separate areas of cerebrospinal fluid and gray and white matter; this segmentation was later applied as a structural template in denoising the fMRI data (see below). Finally, the data were normalized to the Montreal Neurological Institute (MNI) anatomical space (Colin template; http://brainweb.bic.mni.mcgill.ca/brainweb/); the parameters derived from the normalization of T1 images were applied for the normalization of functional images. The normalized voxels were 3.75 × 3.75 × 3 mm^3^ in size. Functional images were smoothed with a 6‐mm full‐width at half‐maximum isotropic Gaussian kernel. According to the estimates of the head motion (realignment step), the maximum translational head motion was <1.5 mm (that is, less than half the voxel size) in all subjects. The time series of translational motion did not differ between the CRPS patients and the healthy subjects (mean ± SD 0.17 ± 0.07 mm vs. 0.17 ± 0.10 mm respectively, *t* = 0.02, *p* = .98, two‐tailed independent samples *t*‐test). For anatomical validation of the preprocessing, see Supplementary methods S1.

#### 2.3.2 Seed‐based analyses

##### Regions of interest

**FIGURE 2:**
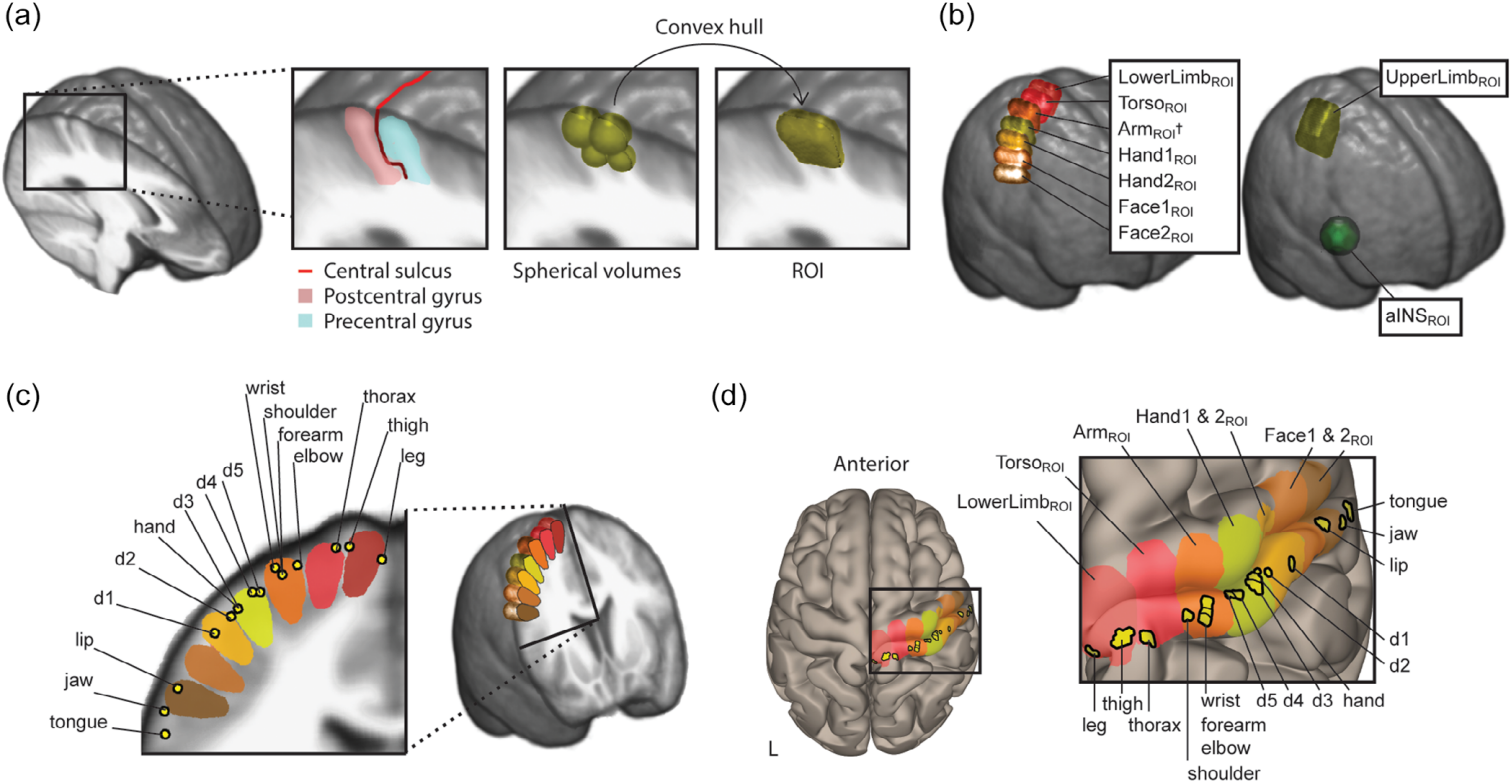
The preparation of the SM1 ROIs. (a) For each ROI we first formed one spherical volume (*r* = 6 mm) at the postcentral gyrus convexity and then its adjacent counterpart at the precentral gyrus convexity. Right below them, approximately perpendicular to the brain surface, we situated two smaller adjacent spherical volumes (*r* = 4 mm). The ROI was then created as a convex hull of these four adjacent volumes. (b) The seven similar sized adjacent ROIs covered the SM1 from its dorsomedial to ventrolateral parts. These ROIs are displayed as 3D renders on the left. A larger ROI with representation areas of the whole upper limb (UpperLimb_ROI_) was created as a convex hull of the three appropriate smaller ROIs (Arm_ROI_, Hand1_ROI_, and Hand2_ROI_). This ROI together with the ROI of the right anterior insula (aINS_ROI_) are displayed as 3D renders on the right. (c) We situated the seven ROIs so that each covered only the mean coordinates of one anatomically distinct body part, described in Roux et al. (2018). These body‐part coordinates are projected onto an oblique brain cutout including the ROIs. The cutout is following approximately the direction of the central sulcus and is perpendicular to the anterior–posterior axis of the seven ROIs. (d) The body‐part coordinates (1 × 1 × 1 mm) and ROIs are displayed as their cortical projections on a template brain. All the 3D brain renders and cutouts in this figure are created with MRIcroGL from a mean whole‐group T1 image, except on (c) the brain‐render is created with CONN toolbox. ROI, region of interest; SM1, primary sensorimotor cortex.

For the seed‐based analyses, we delineated 14 regions of interest (ROIs) in the SM1 and one in the right aINS as follows. First, to study FC within the SM1, we manually split the right SM1 into seven similar sized ROIs (mean volume 2.4 ± SD 0.2 cm^3^) with Mango software (http://ric.uthscsa.edu/mango/). These seven adjacent ROIs covered the SM1 from its dorsomedial to its ventrolateral parts, and each ROI was designed to include a representation area of an anatomically distinct body part, according to the MNI coordinates reported by Roux et al. (2018): the lower limb (referred hereafter as LowerLimb_ROI_; centroid *x* = 9, *y* = −35, *z* = 72), the torso (Torso_ROI_; 18, −39, 70), the upper limb from the shoulder to the wrist (Arm_ROI_; 29, −28, 67), two ROIs for the hand (Hand1_ROI_ and Hand2_ROI_; 37, −23, 60 and 44, −20, 55), and two ROIs for the lips and face (Face1_ROI_ and Face2_ROI_; 48, −14, 47 and 53, −9, 39). We mirrored the ROIs on the right SM1 by the brain's midline to create the corresponding left SM1 ROIs. Figure 2 visualizes the preparation and location of the right SM1 ROIs that, with their contralateral counterparts, were applied as seeds in the seed‐to‐seed analyses. For details of ROI delineations and their validation with anatomical landmarks and Neurosynth meta‐analysis (https://neurosynth.org/), see Supplementary Methods S1. We note that the applied ROIs can only be approximations of the representation areas that in reality do not have strict boundaries.

Second, to study the FC of the representation area of the upper‐limb with the rest of the brain in seed‐to‐voxel analysis, the ROIs Arm_ROI_, Hand1_ROI_, and Hand2_ROI_ were fused together to form a single UpperLimb_ROI_ in each hemisphere (centroid at ±35, −23, 56; volume 9.3 cm^3^).

Third, for the right aINS, we created a spherical ROI (aINS_ROI_) with a radius of 12 mm (center at 44, 8, 0, and volume 7.2 cm^3^, in agreement with [Kim et al., 2013]; Figure 2b). This ROI was selected because the right aINS is thought to be more involved in saliency detection than its left‐hemisphere counterpart (Kann et al., 2016).

All the SM1 ROIs are available for download at https://identifiers.org/neurovault.collection:13334.

##### Group comparisons

In addition to the full‐group comparisons (17 CRPS patients vs 19 healthy control subjects), we performed two selective analyses to minimize and explore the nuisance effects caused by the heterogeneous lateralization of pain in our patient sample: (1) full‐group comparison with flipped data for the five patients with left upper‐limb CRPS and for five age‐, sex‐, and scanner‐matched healthy control subjects (17 vs. 19), and (2) a subgroup comparison including only the patients with right upper‐limb CRPS (12 vs. 18; all females).

##### CONN toolbox

We performed all the seed‐based analyses with standard protocols and tools of the SPM‐compatible CONN toolbox v.18a (http://www.nitrc.org/projects/conn; Whitfield‐Gabrieli & Nieto‐Castanon, 2012) or, when stated below, with Statistical Package for the Social Sciences (SPSS) v.20 software on the FC data extracted from CONN.

Before calculating FC, the CONN toolbox de‐noised the fMRI data by removing with linear regression temporal confounds due to head motion (the realignment parameters from the preprocessing step) and physiological noise estimated with the CompCor method implemented in the CONN toolbox (five components from the principal component analysis of white‐matter and cerebrospinal‐fluid fMRI data; Behzadi et al., 2007). The fMRI data were band‐pass filtered from 0.008 to 0.09 Hz. All the analyses were limited to gray‐matter voxels by using a corresponding SPM8 mask with a threshold of 0.3.

##### Seed‐to‐seed analyses

In the first, subject‐level, seed‐to‐seed analyses, the temporal correlations (*r*_FC_) between the average BOLD signals of selected seeds were calculated subject‐wise. The correlation values were then subjected to Fisher transformation before the second, group‐level, analysis. We studied group FC differences (1) between the SM1 ROIs and (2) between aINS_ROI_ and SM1 ROIs. For the former, we analyzed the FC between each pair of the 14 SM1 ROIs (91 comparisons) using CONN toolbox general linear modelling (GLM) implementation, with *t*‐statistics at the second level. We applied false discovery rate (FDR) corrections for multiple comparisons with a statistical significance level of *q* < .05. For the latter, we analyzed the FC data between the aINS_ROI_ and each of the 14 SM1 ROIs with SPSS to enable flipping only the SM1 ROIs and not the aINS_ROI_. In this analysis, we applied repeated measures 2 × 2 × 7 (GROUP × SIDE × SM1 ROIs) multivariate analysis of variance (MANOVA), and for the effects of disease duration, MSSI and pre‐experiment pain in the patient group, we applied repeated measures 2 × 7 (SIDE × SM1 ROIs) MANOVA.

Seed‐based analyses displayed in the patient group a robust reduction of interhemispheric homotopic FC between the SM1 upper‐limb areas (see Section 3), which we then explored further in post hoc correlation analyses with clinical features. Given the role of the SM1 in encoding the intensity and location of somatic sensations, we reasoned that the reduced FC could be associated with either the intensity or spatial extent of the patients' pain, or both. As a measure of the intensity of pain, we used the patients' pre‐experiment pain ratings (mean ± SD NRS 5.6 ± 1.7; see Section 2.2). The spatial extent of pain was calculated from patients' pain drawings as the percentage of area marked as painful vs the whole template area (19% ± 13%; visualized in Figure 1); these calculations were made separately for the hand, from the corresponding hand template drawings, and for the more proximal upper‐limb areas from wrist to shoulder, from the whole‐body template drawings. In the final analyses, the distal (hand) part was given a weighting of 2/3 and the proximal upper‐limb part 1/3 to account for their relative representation areas in the SM1, which was also reflected in the number of ROIs for these areas (Hand1_ROI_ + Hand2_ROI_ vs. Arm_ROI_). As the measure of the combined effect of pain intensity and extent on FC, we used “pain load,” the product of intensity and extent (calculated after normalization of each measure to a range of 1–10). We analyzed the effects of pain intensity, extent, and load, as well as of disease duration and motor symptom severity, on the interhemispheric FC with partial correlation in SPSS, while controlling for nuisance covariates (see Section 2.3.4).

##### Seed‐to‐voxel analysis

In the seed‐to‐voxel analysis (Biswal et al., 1995), we analyzed group differences separately for the right and left UpperLimb_ROI_. At the first (subject) level, temporal correlations were calculated between the average BOLD signal in the seed region and the BOLD signals of each voxel in the gray matter. At the second level of group‐fMRI random‐effects GLM analysis, Fisher‐transformed correlation values were compared between the groups. Voxel‐level statistical significance was set at *p* < .001 (uncorrected), together with cluster‐level FDR‐corrected *q* < .05. In addition, as the main analysis of the patient group displayed, intriguingly, increased FC between the left UpperLimb_ROI_ and an area in the periaqueductal gray matter (PAG), we used PAG as a seed area in a post hoc seed‐to‐voxel analysis.

We analyzed the effects of disease duration, MSSI and pre‐experiment pain on the FC in the patients with CONN toolbox GLM implementation. All seed‐to‐voxel analyses were limited to brain areas that showed abnormal FC in the patient group in the corresponding group‐comparison analyses.

#### 2.3.3 Independent component analysis

To study the functioning of the SMN, we applied a model‐free data‐driven method, the independent component analysis (ICA) as implemented in FSL (https://fsl.fmrib.ox.ac.uk/fsl/fslwiki/FSL) on our fMRI data preprocessed with SPM8 and de‐noised with CONN. First, we used FSL's MELODIC tool to decompose the fMRI data of 34 subjects (for both patients and the healthy control subjects, *n* = 17) into 30 group‐level components, using a temporal concatenation approach. Next, we applied the dual‐regression method, which provided subject‐wise spatial maps of beta values describing how strongly each voxel of each subject was associated with each of the 30 group‐level components. We visually identified the group‐level SMN from these components (first component) and subjected the corresponding subject‐wise maps to group comparisons with GLM analysis. The probability of group differences was assessed by permutation testing (5000 permutations). We applied threshold‐free cluster enhancement to correct for multiple comparisons with a threshold of statistical significance of *p* < .05.

To study the alterations in the SM1 representation areas of the affected versus the contralateral (“healthy”) limb, we compared, using GLM in SPSS, the subject‐wise dual‐regression mean beta values from the appropriate UpperLimb_ROI_s between patients and healthy subjects. We also applied partial correlation in SPSS to study the effects of clinical characteristics on the UpperLimb_ROI_.

#### 2.3.4 Nuisance effects

For all analyses, we applied age, scanner (GE/Siemens), and percentage of eyelid closures (PERCLOS, see below) as nuisance covariates, and additionally, if applied, flipping of the fMRI data (yes/no).

##### Percentage of eyelid closures

The level of alertness can affect the FC of the sensorimotor and other brain networks (Martuzzi et al., 2010; Tagliazucchi & Laufs, 2014; Tsai et al., 2014). Although instructed to stay awake, subjects may frequently fall asleep during the resting‐state fMRI measurements (Tagliazucchi & Laufs, 2014). Thus, it is important to monitor alertness during resting‐state fMRI (Power et al., 2014), especially in studies addressing disease effects (Tagliazucchi & Laufs, 2014). As drowsiness is associated with eyelid closures, we calculated the PERCLOS (Dinges et al., 1998) during the whole fMRI scan and analyzed group differences with the Mann–Whitney *U*‐test. We applied PERCLOS as a covariant in fMRI analysis to account for effects of drowsiness on FC. See Supplementary Methods S1 for the details of calculating and analyzing PERCLOS.

##### Hand movements

Hand movements also influence the SM1's FC. Importantly for our hypothesis, synchronized bimanual hand movements would increase interhemispheric FC between the M1 hand areas, whereas unilateral hand movements would decrease it (Gabitov et al., 2019; Meister et al., 2010). Thus, to estimate this nuisance effect, we analyzed accelerometer data for possible group differences in the synchrony and amount of left‐ and right‐hand movements with the Mann–Whitney *U*‐test. For the temporal correlation analysis (synchrony), we calculated the accelerometer magnitude vector $a_{m}$, to index hand movement at each time point separately for both hands, with the following formula:

$$a_{m}=\sqrt[{}]{{a_{x}}^{2}+{a_{y}}^{2}+{a_{z}}^{2}}$$

where $a_{x}$, $a_{y}$, and $a_{y}$ are the recorded accelerometer signals in three orthogonal spatial dimensions. As an index of the total amount of hand movements, we applied the mean power of the magnitude vector. To reduce signal artifacts (e.g., slow frequency drifts, MR scanner noise), we filtered the raw accelerometer data to a frequency band of 0.1–8 Hz, which includes the majority of voluntary hand movements (Lee et al., 2019; Wade et al., 2014). For two patients, accelerometer data were corrupted, and thus these analyses were performed for 15 CRPS patients and 19 healthy control subjects.

## 3 RESULTS

We found three major abnormalities in our CRPS type 1 patients compared with healthy control subjects: (1) reduced interhemispheric FC between SMI upper‐limb areas, documented by both seed‐to‐seed and seed‐to‐voxel analyses, (2) increased FC of the SMI cortex with PAG and the right aINS, and (3) disconnection of the SMI upper‐limb areas from the rest of the SMN.

Before describing our results in detail, we want to note that it is unlikely that the observed abnormalities in fMRI‐detected FC would reflect trivial differences between the groups, for example in vigilance levels or in the amount or timing of inadvertent hand movements. To rule this out, we first demonstrated that drowsiness—quantified by the percentage of eyelid closure during the fMRI recording—did not differ between patient and control groups. Second, we found that the amount and synchrony of inadvertent left‐ and right‐hand movements—monitored with accelerometer recordings—did not differ between the groups. Supplementary Tables S1 and S2 show these results in detail.

### 3.1 Reduced interhemispheric FC between upper‐limb SM1 cortices

**FIGURE 3:**
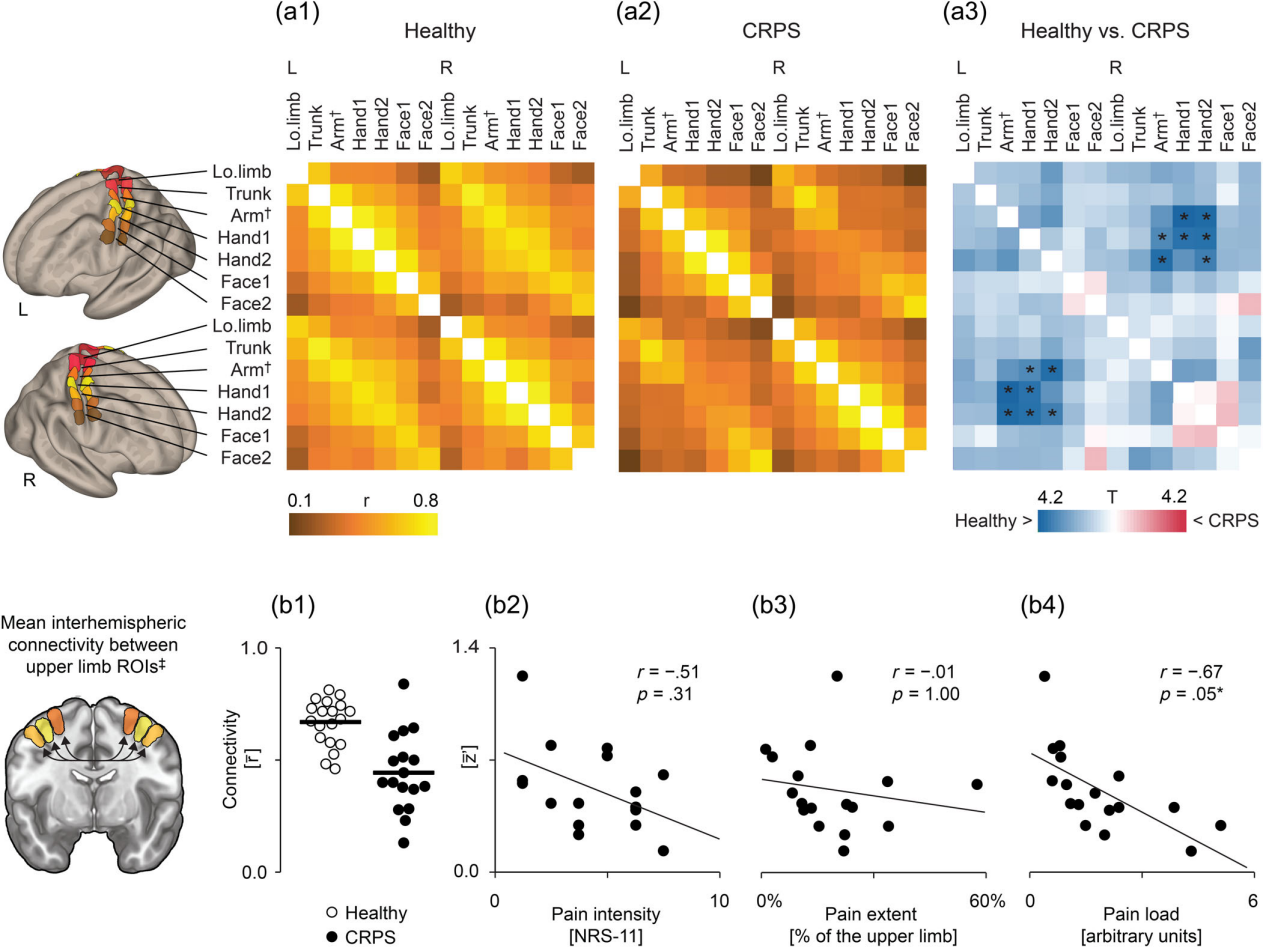
Results of the seed‐to‐seed functional connectivity analysis of the primary sensorimotor cortex. Upper panel: (a1) Pair‐wise functional connectivity correlation matrices for the 14 ROIs on the sensorimotor cortex (seven on each hemisphere) displayed separately for healthy control subjects and (a2) CRPS patients. (a3) The corresponding *t*‐values of the between‐groups analysis. Lower panel: (b1) The individual mean interhemispheric functional connectivity between the six upper‐limb ROIs (three on each hemisphere) for healthy subjects (white circles) and CRPS patients (black circles). (b2) The mean interhemispheric functional connectivity between upper‐limb ROIs of individual patients plotted as a function of pain intensity, (b3) pain extent, and (b4) pain load (intensity × extent). All data were corrected for the nuisance factors of age, MRI, and PERCLOS. †, Arm_ROI_ including the representation area of the upper limb from wrist to shoulder; ‡, Arm_ROI_, Hand1_ROI_ and Hand2_ROI_; CRPS, complex regional pain syndrome; L, left; Lo.limb, lower limb; PERCLOS, percentage of eyelid closures; *r*, Pearson's correlation; R, right; $\bar{r}$, mean correlation between the upper‐limb seeds; ROIs, regions of interest; $\bar{z^{\prime }}$, Fisher transform of $\bar{r}$; T, *t*‐value; *p < .05 (corrected for multiple comparisons).

In the CRPS patients, the interhemispheric FC between the SM1 upper‐limb areas was statistically significantly reduced compared with healthy subjects in both seed‐to‐seed and seed‐to‐voxel analyses. Figure 3 displays the results of the *seed‐to‐seed analysis* that assessed the FC between the SM1 representation areas of different body parts. Here, the group differences manifested specifically between the left and right hemisphere upper‐limb ROIs (seven out of nine interhemispheric ROI pairs; mean ± SD *r*_FC_ = 0.44 ± 0.21 vs. 0.67 ± 0.13; Figure 3, upper panel and Figure S1). In the patients, the mean interhemispheric FC between the upper‐limb ROIs correlated negatively with the pain load (i.e., pain intensity × extent, see Section 2; *r* = −.67, *p* < .05; Figure 3, lower panel), but not with pain intensity alone, pain extent alone, duration of CRPS, or MSSI (see Section 2). The results were similar when the data of the left‐sided CRPS patients were flipped, as well as when only the subgroup analysis of right‐sided CRPS patients was studied (Figure S1).

**FIGURE 4:**
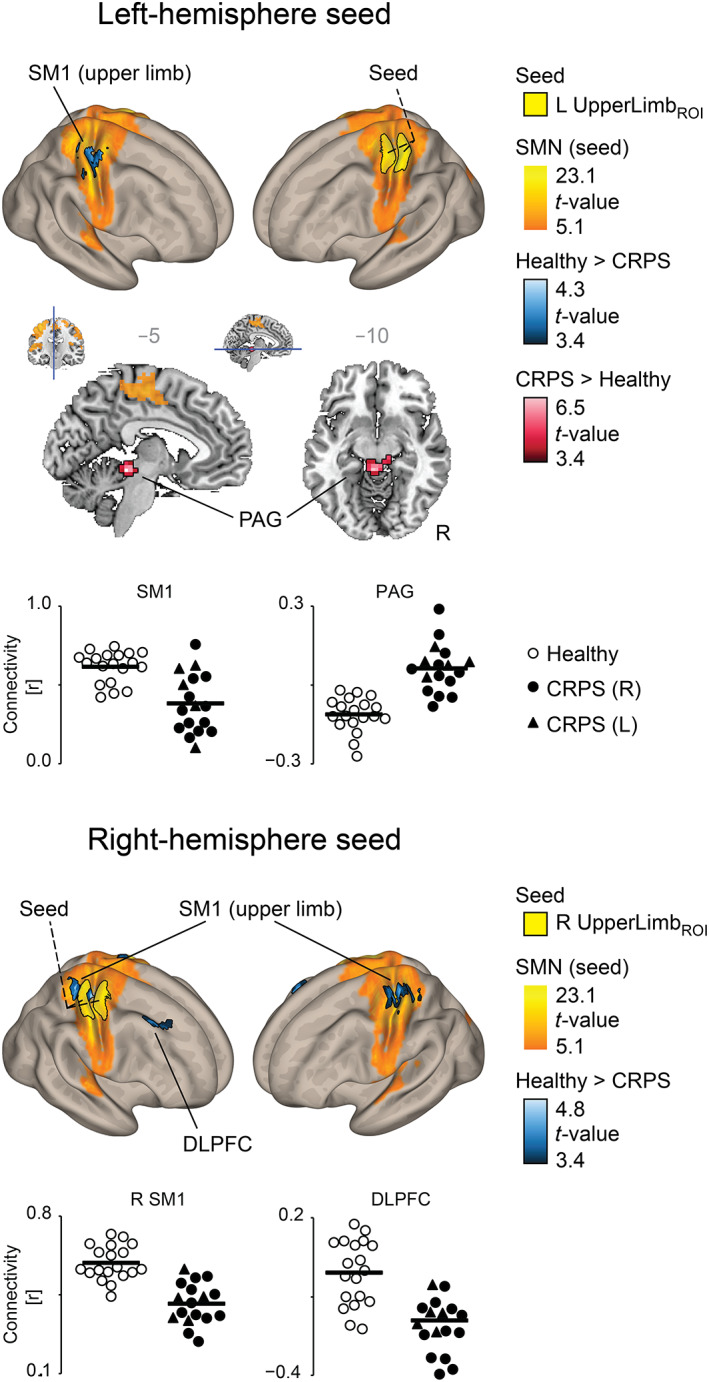
Results of the seed‐to‐voxel functional connectivity analysis for the left and right primary sensorimotor cortex upper‐limb ROIs (upper and lower parts of the figure, respectively) displayed on top of a 3D semi‐inflated brain white‐matter template. The black‐bordered yellow areas indicate the seeds. The statistically significant group differences are color‐coded (black‐to‐blue gradient for decreased and black‐to‐red gradient for increased functional connectivity for patients, compared with healthy subjects). The SMN is displayed with orange‐to‐yellow gradients, based on the thresholded *t*‐values of the healthy subjects. The subject‐wise functional connectivity values are plotted for selected clusters; white‐centered circles represent healthy subjects, black‐centered circles right‐sided CRPS patients, and black‐centred triangles left‐sided CRPS patients. All results were corrected for the nuisance factors of age, MRI, and PERCLOS. CRPS (L), left‐sided CRPS; CRPS (R), right‐sided CRPS. CRPS, complex regional pain syndrome; DLPFC, dorsolateral prefrontal cortex; PAG, periaqueductal gray matter; ROIs, regions of interest; SM1, primary sensorimotor cortex; SMN, sensorimotor network.

Figure 4 and Table 2 display the *seed‐to‐voxel analysis* assessing FC of the upper‐limb SM1 areas across the whole brain. In agreement with the above seed‐to‐seed analysis, the patients (compared with the healthy subjects) displayed reduced FC between the homotopic left and right SM1 upper‐limb areas. In addition, patients showed reduced FC between the right UpperLimb_ROI_ and the right dorsolateral prefrontal cortex (DLPFC). For the group‐specific seed‐based FC maps, see Figure S2. In the subgroup analysis including only patients with right sided CRPS, the statistically significant abnormalities were limited to the reduced interhemispheric FC between SM1 upper limb areas (Table S3 and Figure S3A,B).

**TABLE 2:** Results of the seed‐to‐voxel analyses.

Analysis		Results							
		Cluster					Peak		
Seed	Type	Area	Size (mm^3^)	P‐FDR	Functional connectivity^a^ (*r* mean ± SD)		*x*, *y*, *z* (MNI)	*T*	P‐unc.
					CRPS	Healthy			
L UpperLimb_ROI_	Healthy > CRPS	R SM1	2489	0.003	0.38 ± 0.18	0.62 ± 0.11	30, −35, 59	4.3	<10^−4^
	CRPS > Healthy	PAG	1688	0.031	0.06 ± 0.09	−0.11 ± 0.07	−4, −35, −10	6.5	<10^−6^
R UpperLimb_ROI_	Healthy > CRPS	L SM1	3038	0.001	0.35 ± 0.16	0.59 ± 0.10	−30, −28, 59	−4.7	<10^−4^
		R SM1	1055	0.038	0.40 ± 0.09	0.58 ± 0.08	26, −28, 53	−4.8	<10^−4^
		DLPFC	1055	0.038	−0.20 ± 0.10	−0.02 ± 0.12	30, 21, 50	−4.4	<10^−4^
PAG^b^	CRPS > Healthy	L SM1	11,813	<10^−6^	0.08 ± 0.08	−0.11 ± 0.08	−53, −16, 50	6.8	<10^−6^
		SMA	3206	<10^−3^	0.09 ± 0.10	−0.10 ± 0.11	0, −20, 50	5.1	<10^−5^
		R SM1	3122	<10^−3^	0.06 ± 0.10	−0.12 ± 0.10	38, −35, 62	4.6	<10^−4^
		R SM1	3080	<10^−3^	0.06 ± 0.10	−0.12 ± 0.08	56, −20, 53	5.6	<10^−5^

Abbreviations: DLPFC, dorsolateral prefrontal cortex; FDR, false discovery rate; L, left; M1, primary motor cortex; MRI, magnetic resonance imaging; PAG, periaqueductal gray matter; PERCLOS, percentage of eyelid closures; *r*, Pearson's correlation coefficient; R, right; SM1, primary sensorimotor cortex; SMA, supplementary motor area.

^a^ Corrected for nuisance factors of age, MRI, and PERCLOS.

^b^ Post hoc analysis.

In the seed‐to‐voxel GLM analyses, none of the factors we studied (intensity of pain, duration of CRPS, or severity of motor symptoms) correlated statistically significantly with the patients' abnormal UpperLimb_ROI_ FCs.

### 3.2 Increased connectivity of SM1 with PAG and the right aINS

In the seed‐to‐voxel analysis, the CRPS patients displayed increased FC between the left SM1 UpperLimb_ROI_ and the PAG (Figure 4, upper panel and Table 2). In the post hoc seed‐to‐voxel analyses with the PAG‐area as the seed, FC was statistically significantly increased with both left and right SM1 upper‐limb areas, and also bilaterally with the supplementary motor area (SMA; Table 2 and Figure S4A). In the seed‐to‐voxel sub‐analysis including only the 12 patients with right‐sided CRPS, the FC increase was statistically significant only for the left SM1 upper‐limb area, that is, the representation area of the CRPS‐affected limb (Table S3 and Figure S4B). To explore if PAG‐SM1 connectivity is lateralized depending on the CRPS side, we performed a seed‐to‐seed analysis which showed no statistically significant difference between the FC of PAG with UpperLimb_ROI_s corresponding to either CRPS‐affected or healthy limb (*n* = 17, mean ± SD r_FC_ = 0.10 ± 0.10 vs. 0.07 ± 0.14 respectively, *F*_1,12_ = 1.2, *p* = .29, *η*_p_^2^ = 0.09).

**FIGURE 5:**
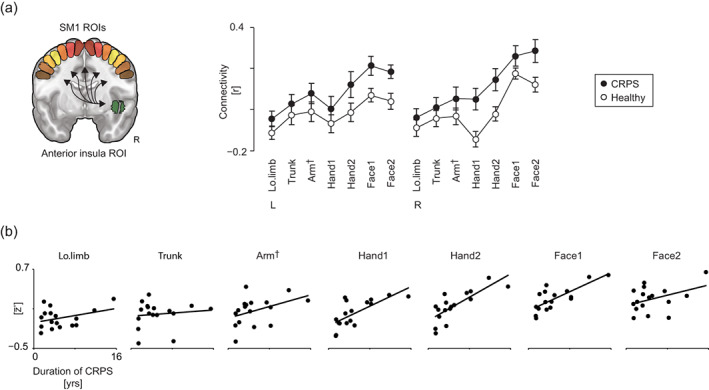
Functional connectivity between the right anterior insula and the primary sensorimotor cortex in the seed‐to‐seed analysis. Upper panel, left: The locations of the ROI of anterior insula and the 14 ROIs of the sensorimotor cortex (7 on each hemisphere). Upper panel, right: The mean ± SEM functional connectivity for healthy control subjects (white circles) and CRPS patients (black circles). Lower panel: Individual functional connectivity values of CRPS patients for each of the seven ROIs of the sensorimotor cortex (averaged across left and right hemispheres) as a function of the duration of CRPS. All data are corrected for the nuisance factors of age, MRI, and PERCLOS. CRPS, complex regional pain syndrome; L, left; MRI, magnetic resonance imaging; R, right; *r*, Pearson's correlation; ROIs, regions of interest; $\bar{z^{\prime }}$, Fisher transform of $\bar{r}$; †, Arm_ROI_ including the representation area of the upper limb from wrist to shoulder.

Figure 5 visualizes the results of seed‐to‐seed analysis focusing on FC between the right aINS and the SM1. The patient group, compared with the healthy subjects, showed statistically significantly increased FC between aINS_ROI_ and the SM1 ROIs (*F*_1,31_ = 5.6, *p <* .05, *η*_p_^2^ = 0.15; see Figure 5, upper panel), with no statistically significant effect by the hemisphere or any specific SM1 ROI. In the additional analysis with flipped data for the left‐sided CRPS patients, and in the subgroup analysis of the 12 right‐sided CRPS patients, the group differences were concordant (*F*_1,30_ = 6.4, *p <* .05, *η*_p_^2^ = 0.18 and *F*_1,25_ = 7.9, *p <* .05, *η*_p_^2^ = 0.24 respectively; see Figure S5).

The longer the CRPS had lasted, the stronger was the FC between the aINS_ROI_ and the SM1 ROIs (main effect *F*_1,12_ = 6.0, *p <* .05, *η*_p_^2^ = 0.33); post hoc analysis of significant interaction effects indicated that this general effect derived especially from FC with the bilateral Hand1_ROI_, Hand2_ROI_, and Face1_ROI_ (*F*_1,12_ = 10.8, *p* < .05, *η*_p_^2^ = 0.47; *F*_1,12_ = 12.6, *p* < .05, *η*_p_^2^ = 0.51; *F*_1,12_ = 11.4, *p* < .05, *η*_p_^2^ = 0.49 respectively; see Figure 5, lower panel). No other clinical characteristics showed statistically significant effects on the FC between ROIs in aINS and SM1.

### 3.3 Upper‐limb SM1 is disconnected from the rest of the SMN

**FIGURE 6:**
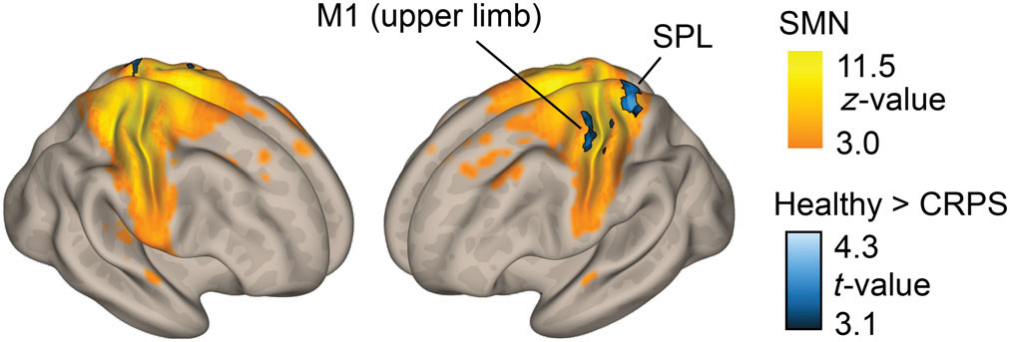
Results of the independent component analysis (ICA) between the sensorimotor network and other brain regions, displayed on top of a 3D semi‐inflated brain white‐matter template. The black bordered black‐to‐blue gradient indicates statistically significantly decreased functional connectivity in CRPS patients compared with healthy subjects. The sensorimotor network, displayed with orange‐to‐yellow gradient, is derived from the data of the healthy control subjects; the thresholds for *z*‐values were chosen by visualization purposes. All results were corrected for the nuisance factors of age, MRI and PERCLOS. ICA, independent component analysis; M1, primary motor cortex; MRI, magnetic resonance imaging; PERCLOS, percentage of eyelid closures; SMN, sensorimotor network; SPL, superior parietal lobule.

Figure 6 shows that in the ICA the patients, compared with healthy subjects, displayed statistically significantly reduced FC between the whole SMN and the left M1 hand area, as well as with the left superior parietal lobule (Figure 6 and Table 3). In the subgroup analysis of the 12 right‐sided CRPS patients, FC was also reduced for the right primary somatosensory cortex (S1) “healthy” upper‐limb area (Figure S3C and Table S4).

**TABLE 3:** Results of the SMN independent component analysis.

Analysis	Results				
	Cluster		Peak		
	Area	Size (mm^3^)	*x*, *y*, *z* (MNI)	*T*	P‐TFCE
Healthy > CRPS	L SPL	1392	−23, −46, 59	4.3	0.023
	L M1	928	−38, −16, 56	4.1	0.033

Abbreviations: L, left; M1, primary motor cortex; SMN, sensorimotor network; SPL, superior parietal lobe; TFCE, threshold‐free cluster enhancement.

The analyses specifically addressing the relationship of the UpperLimb_ROI_s with the ICA‐identified SMN implied reduced FC for SM1 ROIs corresponding to both the CRPS‐affected limb and the contralateral “healthy” limb (patients vs healthy subjects; *F*_1,30_ = 10.0, *p <* .01, *η*_p_^2^ = 0.25 and *F*_1,30_ = 7.3, *p <* .05, *η*_p_^2^ = 0.20 respectively). This FC did not correlate statistically significantly with pain intensity, pain extent, pain load, CRPS duration, or motor symptom severity.

## 4 DISCUSSION

**FIGURE 7:**
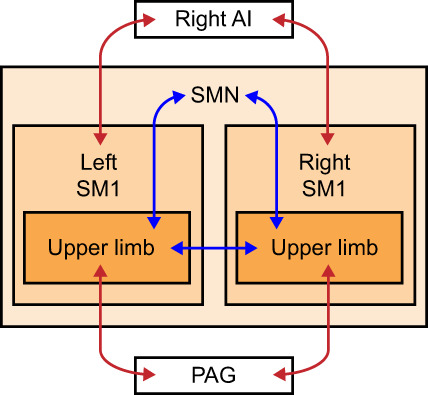
Schematic presentation of the major abnormalities found in functional connectivity in the upper‐limb CRPS patients. The functional connectivity abnormalities in the SM1 were bilateral while the peripheral presentation of CRPS was unilateral. Blue arrows indicate reduced and red increased FC. AI, anterior insula; CRPS, complex regional pain syndrome; FC, functional connectivity; PAG, periaqueductal gray matter; SM1, primary sensorimotor cortex; SMN, sensorimotor network.

Our study on patients with upper‐limb CRPS type 1 revealed multiple alterations in FC of the primary sensorimotor cortex; some of these findings are novel and associated with the clinical features of the patients. Specifically, the patients' interhemispheric FC between homologous upper‐limb SM1 areas was abnormally reduced, corresponding to the peripheral location of the limb pain. This reduction was stronger the higher the patients' spontaneous pain load (intensity x extent of the pain) was. Upper‐limb SM1 areas also displayed reduced FC with the rest of the SMN but increased FC with the PAG. While the patients' whole SM1 showed increased FC with the right aINS, for the hand SM1 this increase was associated with the duration of the syndrome. Figure 7 summarizes the above‐mentioned FC abnormalities.

### 4.1 Interhemispheric FC of SM1 and the asymmetry of sensorimotor functions in CRPS

In healthy humans, the SM1 shows a prominent interhemispheric FC during rest (Stark et al., 2008) that is at its strongest between homologous representation areas (van den Heuvel & Pol, 2010; see Figure 3, upper panel). This homotopic FC can, however, be reduced by injuries at different levels of the nervous system, ranging from peripheral nerves (Hahamy et al., 2015; Liu et al., 2013; Makin et al., 2013) to spinal cord (Hou et al., 2014), and further up to subcortical and cortical brain areas (Park et al., 2011; Xu et al., 2014; Yin et al., 2012) and callosal connections (Roland et al., 2017). The homotopic FC reduction is also clinically relevant as in various unilateral nervous system injuries it is associated with the severity of disuse and sensorimotor dysfunction of the affected limb, as well as with the intensity of persistent pain (Carter et al., 2009; Hahamy et al., 2015; Makin et al., 2013; Xu et al., 2014; Yin et al., 2012). In CRPS patients, the unilateral sensorimotor deficits, limb disuse, and pain are interrelated (Punt et al., 2013). Thus, our finding that the reduced interhemispheric FC in SM1 is correlated with CRPS patients' pain loads and that it is somatotopically specific, is in line with previous studies of unilateral nervous system injuries. However, CRPS type 1 does not, by definition, involve major nervous system injury and so other causes must be considered.

Healthy humans use both hands in everyday life, whereas CRPS patients underuse the affected limb because of their manifold symptoms. In healthy subjects, unilateral limb disuse and sensorimotor deprivation, caused by restricting hand actions by for example a unilateral cast, can induce transient CRPS‐like symptoms in the disused limb (Terkelsen et al., 2008). Along similar lines, disuse of the affected limb predisposes and maintains CRPS (Jänig & Baron, 2004). Consequently, as the brain undergoes limb‐use‐dependent plasticity (e.g., Lissek et al., 2009), part of the abnormal brain function seen in CRPS patients may be associated with limb disuse.

The connectivity of homotopic upper‐limb SM1 areas in healthy persons is related to the symmetry of their bimanual actions. A word of caution is however needed in the interpretation of FC and its alterations in understanding the underlying brain physiology. FC between two brain areas tells that the activity of those areas covaries. Thus, the reduced homotopic FC during asymmetrical bimanual movements compared with symmetrical movements (Meister et al., 2010) and during unimanual movements compared with keeping hands still (Gabitov et al., 2019) are expected because the applied tasks affect SM1 activation asymmetrically and therefore unavoidably reduce FC. However, asymmetrical actions can also produce aftereffects on homotopic FC. The reduction of homotopic FC persists while hands are at rest if the hand use has previously been exceptionally asymmetric for several days because of a unilateral cast. This reduction normalizes during the subsequent cast‐free days (Newbold et al., 2020). In a similar way, resting‐state homotopic FC is also reduced momentarily during transient unilateral sensorimotor denervation, such as supraclavicular peripheral nerve block (Melton et al., 2016). Thus, also our findings could associate with the unilateral sensorimotor deprivation in CRPS patients. Future studies are to show in more detail how the SM1 homotopic FC reduction in CRPS patients reflects asymmetric symptoms, is this FC reduction reversible and/or related to the disease mechanisms.

The fMRI signals of two brain areas could be temporally correlated—resulting in statistically significant

FC values—for different reasons, including synchronous sensory input or communication between the areas through direct or indirect structural pathways. For the strong SM1 homotopic FC, and for normal bimanual coordination as well, interhemispheric signaling through transcallosal neural pathways is especially important (Chettouf et al., 2020; Roland et al., 2017). In healthy persons, the net effects of resting‐state interhemispheric interactions between the SM1 areas are inhibitory, especially the GABAergic ones (Daskalakis et al., 2002; Ferbert et al., 1992; Hlushchuk & Hari, 2006). Although the interhemispheric neural interactions have been addressed in CRPS only through motor performance (Bank et al., 2014; Bank et al., 2015; Berryman et al., 2018), electrophysiological studies in other syndromes, as well as in healthy persons, suggest imbalanced interhemispheric inhibition during asymmetric sensorimotor function (unilateral pain, disuse, overuse, or denervation) (Alhassani et al., 2019; Avanzino et al., 2011; Schabrun et al., 2016; Werhahn et al., 2002). Thus, the observed reduced homotopic FC could in part reflect the imbalance of interhemispheric inhibition.

Decreased homotopic FC in SM1, as well as unilateral interhemispheric inhibitory changes and asymmetric GABA levels are associated with hyperalgesia (Alhassani et al., 2019; Niddam et al., 2021; Schabrun et al., 2016), one of the hallmark signs of CRPS. In CRPS, as in many other “unilateral” syndromes, hyperalgesia and other abnormalities of sensorimotor functions are also expressed to varying degrees in the contralateral “healthy” limb (Dietz et al., 2021; Hotta et al., 2015; Ramalho et al., 2019; Wahren, 1990). In these conditions, the spread of symptoms to the contralateral side may arise through interhemispheric connections (Carson, 2020; Forss et al., 2005). Thus, further studies of the role of interhemispheric interaction in CRPS and of homotopic FC in general could be of clinical importance. Further, targeting the affected hemisphere through interhemispheric interactions (e.g., training of the healthy limb Perez et al., 2007), or balancing the interactions by modulating homotopic FC (e.g., bimanual training [Bank et al., 2015; Meister et al., 2010] or transcranial brain stimulation [Sehm et al., 2013; Stagg et al., 2014; Watanabe et al., 2014]), could serve as novel approaches for interventions in CRPS.

### 4.2 Bilateral disengagement of the upper‐limb SM1 from the rest of the SMN in CRPS

In our CRPS patients, FC was reduced between upper‐limb SM1 (for painful and nonpainful hand) and the distributed SMN. To our knowledge, this is a novel finding in patients with unilateral chronic limb pain. For the SM1 corresponding to the painful limb, the reduced FC may reflect nociception or sensorimotor deprivation affecting the SM1 and other parts of the SMN to different degrees. Similar reduced FC has previously been observed in healthy persons under sustained experimental pain (Kim et al., 2013) as well as in limb amputees without substantial pain (Makin et al., 2015); in the latter case, the likely cause for FC reduction is the denervation of the SM1 cortex. However, these earlier studies did not report disengagement of the contralateral (unaffected) SM1 from the other parts of the SMN, suggesting that such disengagement is unique for CRPS. This disengagement may be related to subclinical symptoms often present in the contralateral limb of a CRPS patient and to the strong interaction between homologous areas in the two hemispheres. Spread of the disease process to the other hemisphere has been documented in a CRPS patient in whom somatosensory responses to tactile finger stimulation elicited bilateral S1 activation, in strong contrast to only contralateral activation in healthy subjects; at the same time, the symptoms had fully spread to the previously healthy contralateral limb (Forss et al., 2005).

One factor contributing to the spread of CRPS symptoms to the originally healthy limb could be SM1 disinhibition that has been demonstrated bilaterally in M1 of CRPS patients as shortened rebounds (enhancements) of the rolandic magnetoencephalographic 20‐Hz activity following tactile stimuli (Juottonen et al., 2002), by reduced suppression of motor‐evoked potentials to paired TMS pulses (Schwenkreis et al., 2003; Strauss et al., 2015), as well as by reduced suppression of the second somatosensory response in S1 to paired median‐nerve stimuli (Lenz et al., 2011). Whether and how these alterations relate to one another will, however, need further research.

### 4.3 FC between SM1 and the right aINS, and the salience of pain in CRPS

We observed increased FC between the right aINS and SM1, with statistically significant dependence on duration of CRPS for the hand SM1. In healthy persons, the aINSs show only weak FC with the sensorimotor cortex (Wiech et al., 2014) but connectivity is strengthened during sustained pain, especially with the SM1 representation area of the painful limb (Kim et al., 2013). Accordingly, increased aINS–SM1 connectivity has been observed previously in various chronic pain syndromes, such as fibromyalgia (Kim et al., 2015; Kutch et al., 2017), chronic back pain (Kim et al., 2019), and chronic pelvic pain (Kutch et al., 2017). In these conditions, the increased FC was associated with the intensity, location, and extent of the pain.

However, in our patients, the increased aINS–SM1 connectivity was not limited to the representation area of the CRPS‐affected limb but appeared for the whole SM1 and maintained the normal heterogeneity over different somatotopic SM1 areas (see Figure 5; Hegarty et al., 2020). This somatotopical nonspecificity suggests contributions from factors other than the noxious input; for example, interoceptive attention and higher pain sensitivity have been associated with enhanced aINS–SM1 coupling (Veréb et al., 2021; Wang et al., 2019). Supporting the effect of attention, a recent study by Kim et al. (2018) showed that CRPS patients have abnormally strong FCs between their attention network and both salience and SMNs. Here, the salience network comprises, in addition to the aINS involved in both the affective pain circuitry and in the detection of salient sensory stimuli (Menon & Uddin, 2010), the temporoparietal junction, mid‐cingulate cortex, and the DLPFC in both hemispheres (Kucyi & Davis, 2015).

In our patient group, the coupling between the upper‐limb SM1 and right aINS was stronger the longer CRPS had lasted. This result fits well with previous reports that longer duration of CRPS is associated with increased pain sensitivity and intensity, as well as with amplification of sensory components of pain experience (Reimer et al., 2016; Schwartzman et al., 2009).

Recently, the altered aINS–SM1 connectivity in CRPS has been hypothesized to affect somatosensory (proprioceptive) perception, for example distorting the body image, although somatosensory input as such is not modified, and the alteration is considered to emerge within the predictive‐coding framework (Kuttikat et al., 2016). In this context, the increased correlation between aINS and SM1 could reflect more intensive communication between these areas, given that the aINS is suggested to coordinate hierarchical processing of tactile prediction errors (Allen et al., 2016).

With these perspectives, increased aINS–SM1 connectivity could be related to a vicious circle where the pain disturbs somatosensory predictions and increases attention to all somatosensory input—including exteroception, proprioception, and interoception—which may then further intensify the pain. However, before complex conclusions are drawn, future research should first firmly establish the strengthening of aINS–SM1 coupling in CRPS and study its nature in more depth.

### 4.4 FC between PAG and SM1 and the endogenous pain modulation in CRPS

We found, beyond our hypotheses, that CRPS patients have abnormal positive FC between the PAG and the SM1 upper limb representation areas (predominantly in the painful limb region) and the SMA. Normally, in healthy persons during rest, both PAG–SM1 and PAG–SMA FCs are negative (see healthy subjects in Table 2; Kong et al., 2010), whereas, during intense pain, these FCs turn positive (akin to our CRPS sample, see Table 2; Linnman et al., 2012). For CRPS, PAG connectivity has not previously been explored, but for many other pain syndromes it has (see e.g., Dahlberg et al., 2018; Pahapill et al., 2020; Wei et al., 2016; Yu et al., 2014). In these studies, if and only if the patients had had a homogeneous location of pain, were the PAG–SM1 and PAG–SMA FCs, specifically, increased compared with healthy persons with or without similar pain. Thus, it appears that the increased FC in PAG–SM1 and PAG–SMA during pain is a normal somatotopic phenomenon, but which may be enhanced in pain syndromes (Dahlberg et al., 2018). Given the role of PAG, this could relate to abnormalities in descending pain modulation. Previously, the latter has been shown to present bilateral alterations although the CRPS was unilateral (Seifert et al., 2009). Moreover, during endogenous modulation of pain in either side, unilateral CRPS patients have shown reduced activity in PAG (Freund et al., 2011). In line with these findings, our PAG–SM1 results included the SM1 upper‐limb areas bilaterally.

Thus, we suggest that the increase in PAG–SMN connectivity in CRPS may reflect altered endogenous pain modulation, which should be of importance in future studies on this area.

### 4.5 Somatotopy in the SM1 and the peripheral manifestation of CRPS

To our knowledge, of studies focusing on SM1 FC in CRPS, ours is the first to show alterations within the SM1 with symptom‐matching somatotopy. Only a few studies on CRPS have explored other brain areas and analyzed seeds outside the SM1 (putamen, ACC, and thalamus) which then showed FC changes with SM1 subareas that matched somatotopically the peripheral CRPS manifestation (Azqueta‐Gavaldon et al., 2019; Di Pietro et al., 2020; Youssef et al., 2019, respectively). Our results do not support these previous findings, which suggest that our SM1 seeds could have been even more specific for the common pain loci across our CRPS patients. In general, homogeneous pain loci within a patient sample are a prerequisite for finding alterations in a common fMRI space. Of the earlier studies in CRPS that focused on the SM1, only van Velzen et al. (2016) had a sample as homogenous as ours (upper‐limb pain with variable distribution). In contrast to our study, Velzen et al. collected their fMRI data while subjects rested with their eyes closed, which may have normalized their SMNs (Martuzzi et al., 2010; Tagliazucchi & Laufs, 2014; Tsai et al., 2014), thus explaining why their ICA revealed no abnormalities for CRPS.

### 4.6 Limitations

Our study has some limitations. First, we had a relatively small sample size (17 patients, 19 control subjects). Second, we collected pain ratings before and not during fMRI measurements, limiting our conclusions on the relationship between pain levels and brain abnormalities. Third, although our seed areas should be valid for group‐comparison purposes (see Supplementary Methods S1), functional localizers would have enabled more accurate study of the relationship between the SM1 reorganization and SMN aberrancy in CRPS patients. In addition, as the somatotopic presentation of different body parts in the SM1 is not even, our approach of dissecting SM1 into seven similar sized ROIs to present five body parts serves only as a crude estimate of the true body presentation areas. Similarly, regarding the localization, given the small size of PAG and the limited resolution of fMRI, it is possible that the cluster we consider encompassing PAG, actually involves some other midbrain structures. Fourth, we had to use two different fMRI scanners and although data from different scanners should be comparable (Forsyth et al., 2014; Gee et al., 2015), as has been specifically shown for the two scanners applied here (Raij et al., 2016), different scanners can increase variability in the data and thereby decrease statistical power. Finally, with an additional sample of lower‐limb CRPS patients, we would have been able to study the somatotopy of the brain alterations more profoundly. The strengths of our study include the careful CRPS diagnostics using recent criteria, multidimensional examinations of the CRPS limbs, and fMRI analyses with careful consideration of confounding factors. However, we must acknowledge the potential heterogeneity of the pathophysiology underlying different patients' CRPS symptoms, but this is a common concern in all studies of CRPS.

### 4.7 Conclusions

In conclusion, our findings of the decreased FC within SM1 and the strengthened FC between SM1 and the aINS and PAG provide novel insights into the pathophysiology of CRPS. Specifically, our results suggest that the chronic pain reshapes the cortical SMN, with somatotopic emphasis. The observed increase of the alterations with disease duration indicates that some of the effects may be secondary to the chronic pain. As the observed FC alterations may be involved in initiation, maintenance, and manifestation of CRPS symptoms, they might have clinically relevance in the prognosis of the disease and in pinpointing targets for intervention.

## AUTHOR CONTRIBUTIONS

Jaakko Hotta contributed to the conception and design of the work, the acquisition, analysis, and interpretation of data and drafting the article. Jukka Saari contributed to the acquisition and analysis of data, as well as to drafting the article. Hanna Harno and Eija Kalso contributed to the conception and design of the work, and the acquisition of data. Nina Forss and Riitta Hari contributed to the conception and design of the work, the interpretation of data and to drafting the article. All authors have contributed to critical revision of important intellectual content of the article and approved the final version.

## CONFLICT OF INTEREST STATEMENT

The authors declare no conflicts of interest.

## SUBJECT CONSENT STATEMENT

Prior to participation, subjects gave written informed consent.

## Supporting information


**DATA S1:** Supplementary methods.

**FIGURE S1:** Supplementary figures.

**TABLE S1:** Supplementary tables.

## Data Availability

The data that support the findings are not available due to privacy/ethical restrictions. All the ROIs in the primary sensorimotor cortex are available for download at https://identifiers.org/neurovault.collection:13334.
