# Supplementary material for: Somatotopic disruption of the functional connectivity of the primary sensorimotor cortex in complex regional pain syndrome type 1
Source: Hum Brain Mapp. 2023 Oct 14;44(17):6258–74. doi: 10.1002/hbm.26513 (PMC10619416; doi:10.1002/hbm.26513)
Supplement: Supplementary file 1 — DATA S1: Supplementary methods. [file HBM-44-6258-s002.docx]

# Supplementary methods

## Preparing the regions of interest

To study the somatotopic order of SM1 connectivity, we divided SM1 into multiple adjacent ROIs and applied them as seeds in our analyses. We created the ROIs using Mango software ([http://ric.uthscsa.edu/mango](http://ric.uthscsa.edu/mango/)) that allows us to create and visualize ROIs of different shapes in the desired MRI space and to conjoin them with a “convex hull” tool.

We determined the locations and extents of ROIs by applying both (1) the mean T1 image of our subject sample and (2) the mean MNI coordinates for the representation areas of sixteen different body parts in Brodmann area 1 (BA1), based on intracranial cortical stimulations in 50 subjects (Roux et al., 2018); we only included body parts that were reported in more than one subject (leg, thigh, thorax, elbow, forearm, shoulder, wrist, fingers 1–5, hand, lip, jaw, tongue). In Mango software, we overlaid the body-part coordinates on top of the mean T1 image and fitted seven spherical volumes (r = 6 mm) next to each other to cover the postcentral gyrus convexity from lower-limb (dorsomedial) region to lip (ventrolateral) area so that each volume included only BA1 coordinates belonging to one distinct anatomical area: 1) lower limb, 2) torso, 3) upper-limb from shoulder to the wrist, 4–5) two volumes for hand and fingers, 6–7) two volumes for face (including lips).

Next, we created for each of these volumes a similar-sized counterpart on the opposite side of central sulcus i.e. at the precentral gyrus convexity. Further, to extend the ROIs more deeply into the gyri, we placed for each of these volume-pairs another pair of spherical volumes (r = 4 mm) below them (approximately perpendicular to the brain surface); all the volumes were placed next to each other. Lastly, we created a convex hull of each set of these four volumes to build the seven ROIs applied as seeds in our analyses. These ROIs were named according to the body-part coordinates they covered: 1) LowerLimb_ROI_, 2) Torso_ROI_, 3) Arm_ROI_ (from wrist to shoulder), 4–5) Hand1_ROI_ and Hand2_ROI_, and 6–7) Face1_ROI_ and Face2_ROI_.

Further, we created a convex hull of the three ROIs covering upper-limb areas (Arm_ROI_, Hand1_ROI_, and Hand2_ROI_), to build one larger ROI corresponding to the whole upper-limb, named as UpperLimb_ROI_. See [Figure](#bookmark) 2 in the main manuscript for the visualization of the preparation and location of these ROIs.

## Validating the regions of interest

We validated the body-part representations included in our ROIs with Neurosynth (<https://neurosynth.org/>) that automatically extracts from a large library of published neuroimaging studies (currently ca. 15,000 articles) brain activation coordinates that are tagged with the corresponding article’s keywords. Thus, with the application, one can perform large-scale meta-analyses with desired keywords to find associated brain activations.

We used four keywords in Neurosynth: *hand* (coordinates from 879 articles), *fingers* (330), *arm* (84), and *foot* (83). For each of these keywords, we performed a meta-analysis, and from the resulting four z-score maps we extracted the z-scores in MNI space separately for each of our 7 bilateral ROIs; the results of the meta-analyses were corrected for multiple comparisons using false discovery rate (FDR) with a threshold of q < 0.01.

Figure M1 shows that for keywords *hand* and *fingers* the average z-scores were highest in the Hand1_ROI_ and Hand2_ROI_ (these two ROIs together included 65% and 67% of the sum of z-scores respectively), for the keyword *arm* in the Arm_ROI_ (30%), and for the keyword *foot* in the LowerLimb_ROI_ (58%). We also explored the coordinates of the peak z-score from the whole brain maps. The peak coordinate for the keyword *hand* was inside the left Hand1_ROI_ (−34, −22, 56), for the keyword *fingers* just at the borderline between the left Hand1_ROI_ and Hand2_ROI_ (−28, −20, 56), for the keyword *arm* a few millimetres anterior to the left Arm_ROI_ (−28, −16, 56), and for the keyword *foot* inside the left LowerLimb_ROI_ (−4, −28, 74). Thus, these results indicate that our ROIs were reasonably located, at least regarding the upper- and lower-limb representations.


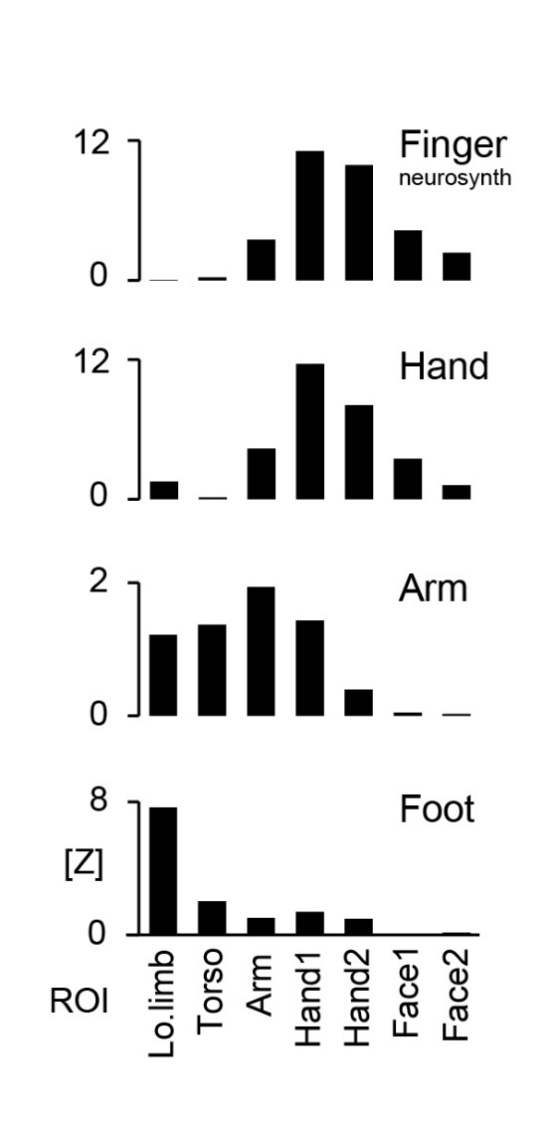


Figure M1. Bar graphs presenting the results of the four Neurosynth meta-analyses as mean z-scores for each of our seven ROIs (x-axis). The keyword for each meta-analysis is presented at the right top corner on each bar graph (finger, hand, arm and foot). Note the variable scale for the z-score in y-axis.

Since we were especially interested in the connectivity of the upper-limb representation areas, we further validated, specifically for our data, the location of the hand ROIs with anatomical landmarks of the hand representation in M1: the hand knob and the hand hook (Yousry et al., 1997). Given the successful normalization of our MRI data, we were able to identify these landmarks from the whole-group mean T1 images. By visual inspection, we could conclude that our Hand1_ROI_ and Hand2_ROI_ covered these landmarks and adjacent postcentral gyri to a great extent, ensuring us with correct locations for the hand seeds for our analyses (see Figure M2).


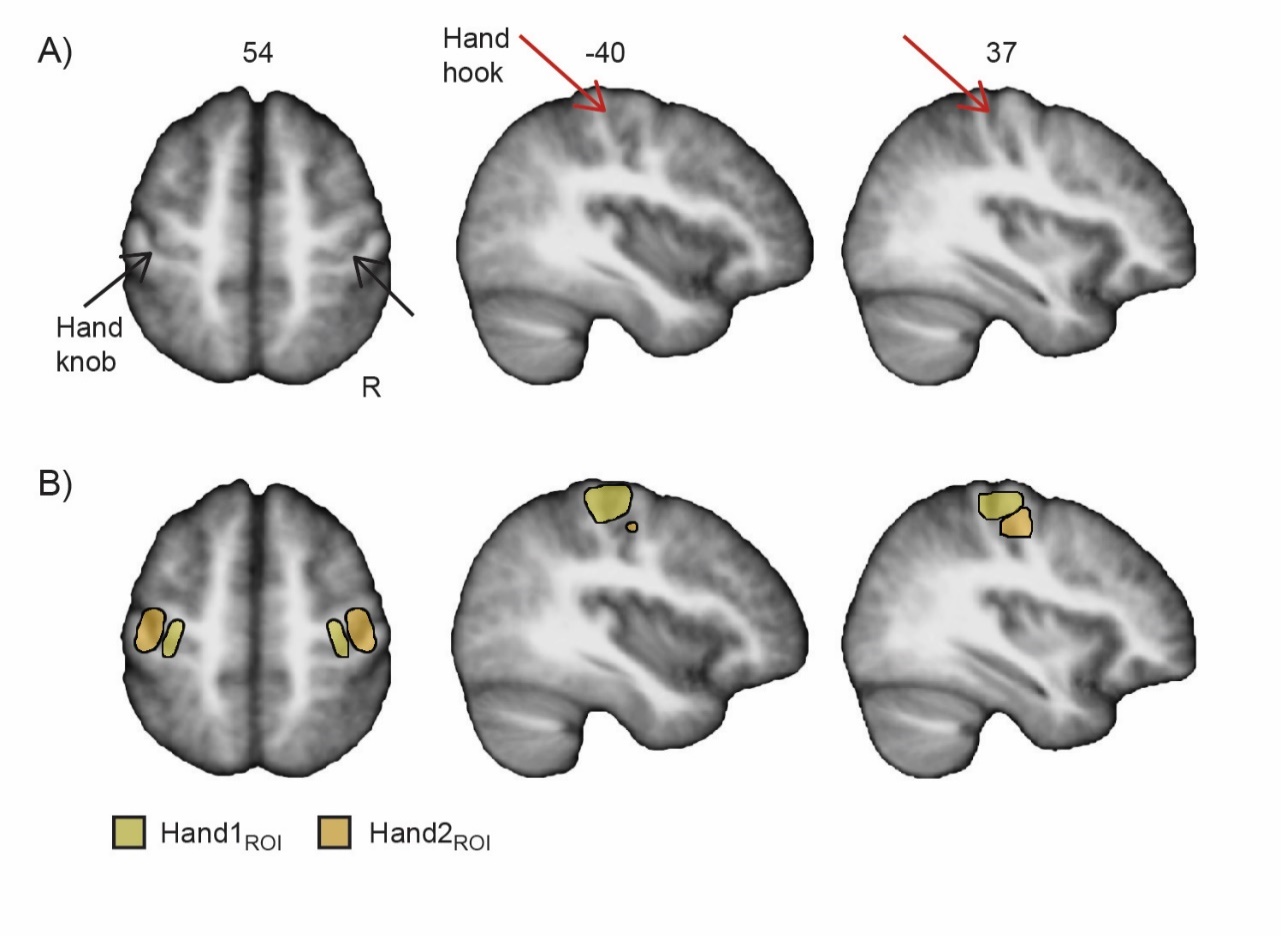


Figure M2. (A) The hand knob (black arrow) could be identified from the axial slice of the whole-group mean T1 images and the hook (red arrow) from the sagittal slices. (C) The seed areas (ochre) covered the hand knob and hook, and adjacent post-central gyrus.

To evaluate how well our hand ROIs covered the hand areas in each individual subject, we explored the locations of the hand knob in each subject. To quantify this, we identified a posterolateral point of the hand knob in all T1 images (see Figure M3A); for individual T1 images, this identification was performed in the native space of the T1 image and the coordinates were converted to the common MNI space using individual normalization parameters (see chapter *Preprocessing* in the main text). According to visual inspection, the knob coordinates were located within the Hand1_ROI_ and Hand2_ROI_ in all subjects. The mean ± SD distances between the mean and individual knob coordinates were 4.6 ± 3.1 mm (range 1.0 – 11.8 mm) in the left hemisphere and 5.3 ± 2.8 mm (1.7 – 13.3 mm) in the right hemisphere. Given the inaccuracy of our visual identification of knob coordinates, we considered these numbers to indicate acceptable variance in the anatomy of the seed area between the subjects. Further, these distances did not differ between the patient and control groups in either the left or right hemisphere (4.8 ± 3.4 mm vs. 4.5 ± 2.9 mm, *p* = 0.75 and 5.0 ± 2.5 vs 5.7 ± 3.0 mm, *p* = 0.47 correspondingly; two-tailed independent-sample *t*-test). The knob’s mean coordinates in the left (−39, −25, 54) were at 6.4 mm distance from the peak coordinate of the Neurosynth meta-analysis for the keyword *hand* and at 5.3mm distance from that of the keyword *fingers*, suggesting successful normalization of the MRI data. See [Figure M](#Ref27572254)3B and C for the visualization of the hand knob areas across subjects.


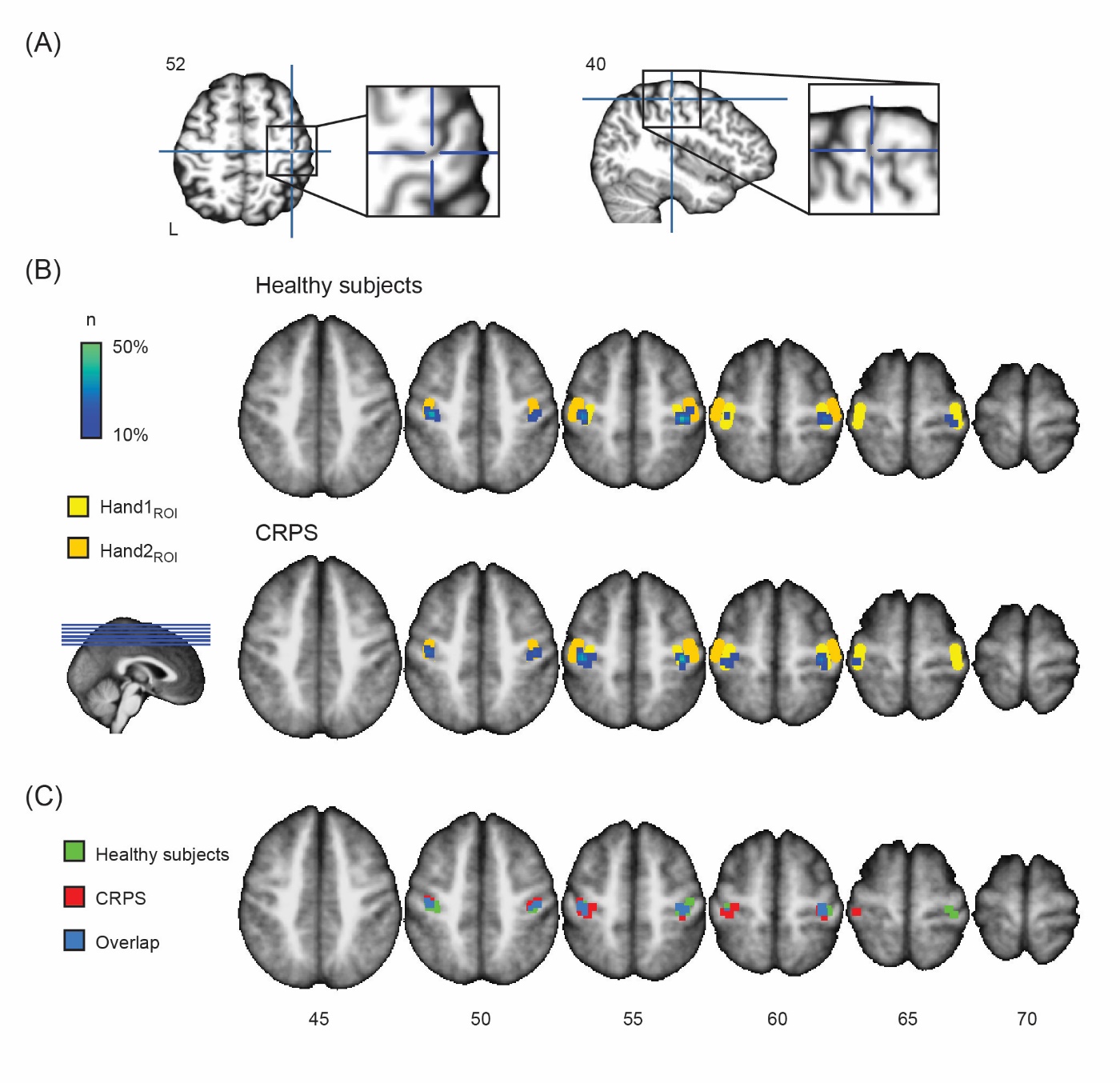


Figure M3. (A) Representative T1 image in its native space, with blue cross-heads marking the hand knob in the axial plane on the left and the hand hook in the sagittal plane on the right. The markers were manually positioned so that they were i) at the surface of the knob, ii) the corresponding axial slice showed the knob at its largest protrusion to the central sulcus and iii) the sagittal slice showed the hand hook. (B) Markers for the hand knob in the CRPS patients and healthy control subjects (blue-green gradient) overlaid on top of the mean T1 image and the ROIs for the hand (yellow and ochre). For visualization purposes, the markers were magnified to a size of 7x7x7mm. The gradient colour indicates the percentage of overlap of the markers in the subject group. (C) The markers for the controls (green) and patients (red) and their overlap (blue).

## Vigilance and percentage of eyelid closures during fMRI

Vigilance is known to affect brain’s functional connectivity. To monitor vigilance, we monitored signs of drowsiness (e.g. prolonged eye-closures, errant fixation, and slow eye movements) from the online video of the eye during the fMRI. Based on the general appearance during the monitoring, we classified each subject into a vigilance class of either 1) alert, 2) tired or 3) sleepy (authors J.H. and J.S.). Additionally, to quantify alertness objectively, we calculated percentage of time that the subjects had their eyes closed during the fMRI (percentage of eyelid closure i.e. PERCLOS), a metric known to increase during the deterioration of vigilance (Abe et al., 2011; Dinges et al., 1998). We calculated PERCLOS for the whole 8-min scan time, excluding blinks (< 500 ms closure; Caffier et al., 2003), and applied it as a nuisance covariant in our fMRI analysis.

In our setting, the PERCLOS could be evaluated either manually from the video recordings (PERCLOS-v) or from the eye-tracker’s pupil-size data as unrecognized pupil (PERCLOS-et). For the calculation of PERCLOS-v, author J.H. manually detected eye closures following the methods described by Dinges et al. (1998), and PERCLOS-et was computed by a home-made code (by author J.S.) in Matlab (https://se.mathworks.com/products/matlab.html). Due to technical issues, some of these data were corrupted or missing; we had successful video recordings from 25 subjects (10 CRPS, 15 healthy) and eye-tracking data from 32 subjects (14 CRPS, 18 healthy).

Since eye-tracker’s pupil recognition is prone to artefacts, we applied PERCLOS-v as a primary measure. For the remainder, we considered PERCLOS-et to be reliable if it was in line with the subject’s pre-estimated vigilance-class, i.e. closer to a mean PERCLOS-v of that vigilance-class than another (5 CRPS, 3 healthy). If not, then we applied the mean PERCLOS-v of subject’s pre-estimated vigilance-class (1 CRPS, 1 healthy), as we did with a patient missing both the video and the eyetracker data. The mean ± SD of PERCLOS-v in the three vigilance-classes were: alert 0.4 ± 0.7 % (n = 19), tired 5.9 ± 0.2 % (n = 4), and sleepy 20.1 ± 4.8 % (n = 3). See Figure M4 for a bar-chart with subject-wise vigilance class and PERCLOS.


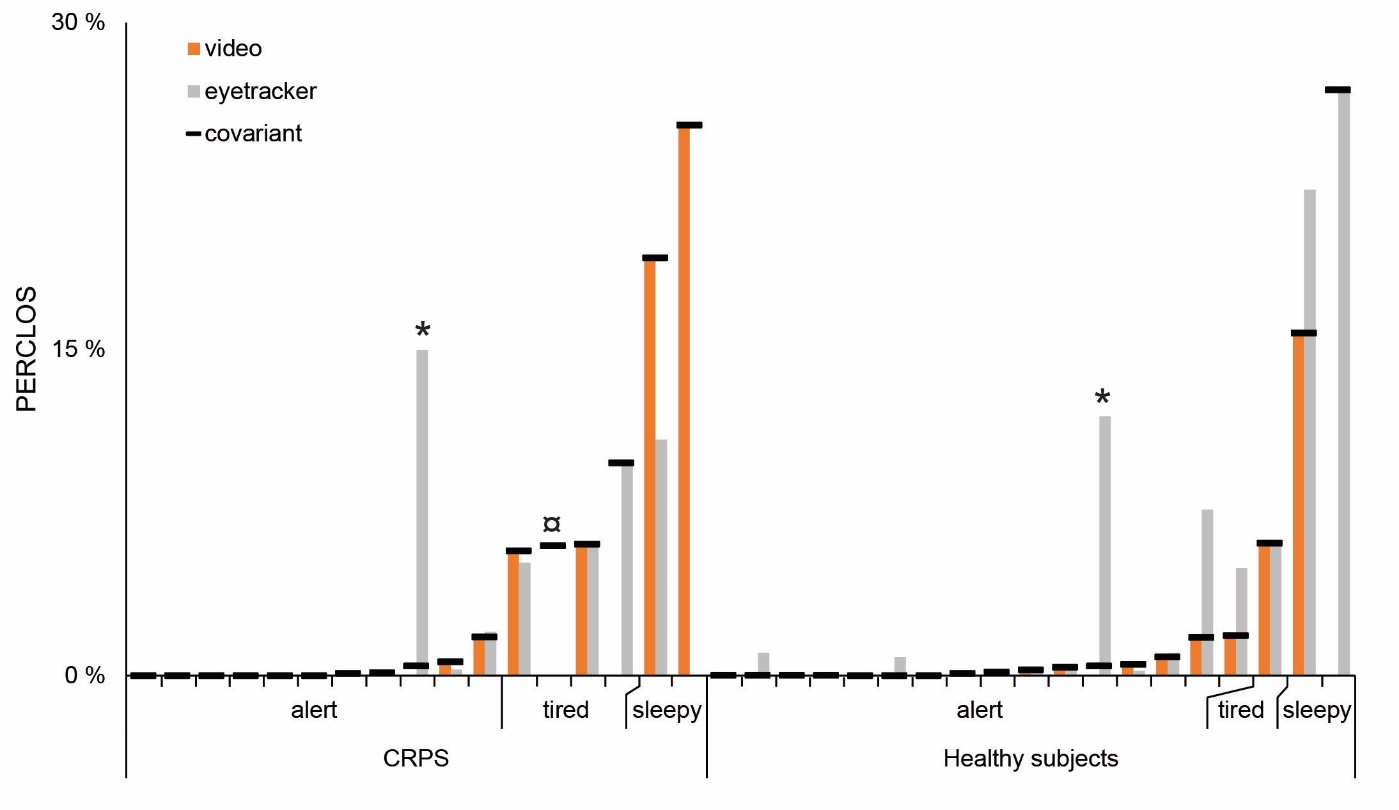


Figure M4. Percentage of eyelid closures (PERCLOS) during the 8-min resting state fMRI scan for each subject. Orange bars present the PERCLOS calculated manually from the video recordings (PERCLOS-v) and gray bars the PERCLOS calculated from the eye-tracker’s pupil recognition data (PERCLOS-et). PERCLOS-v was the preferred method because PERCLOS-et is more prone to artefacts. The black horizontal lines present the PERCLOS values allocated to each subject to be used as a covariant in the fMRI analysis. Two subjects*, who were pre-estimated as alert based on the on-line eye-monitoring during the scan, had unreliable eye-tracker recordings and missing video data. Thus, they were allocated the average PERCLOS-v of the alert subjects. For one subject ¤, who was pre-estimated as tired, both the eye-tracker and video data were missing, and she was allocated the average PERCLOS-v of the other tired subjects.

Although a larger portion of patients appeared in online monitoring as tired or sleepy than in healthy control subjects (35% vs 16%) there was no statistically significant difference in the PERCLOS between the two groups (median 0.4 % and range 0.0–25.3 % vs 0.3 % and 0.0–26.9 % correspondingly, *p* = 0.64, Mann-Whitney U-test).

To evaluate the reliability of PERCLOS-et, we calculated its correlation with PERCLOS-v. To increase the current sample size (n = 24), we calculated both parameters also from another fMRI experiment in which our subjects had participated (n = 22). In overall, PERCLOS-v and PERCLOS-et correlated statistically significantly (r = 0.85, *p* < 1x10^−13^, n = 46: see Figure M5), pairwise difference being 1.5 ± 2.8% (mean ± SD). The difference was in majority of cases from 0% to 3%, but in five sample pairs differences were larger (6% to 13%).


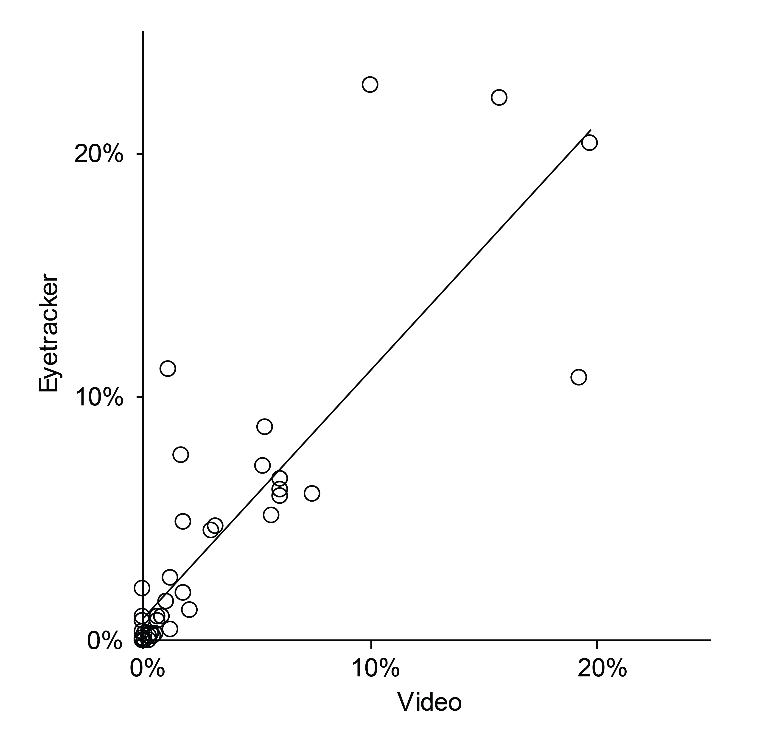


**Figure M5. The percentage of eyelid closures during the fMRI scans calculated from the eye-tracker data as a function of that from the video data. The values are plotted separately for each subject with both eye-tracker and video data available from the resting state fMRI scan (n = 24), and from another fMRI scan performed to same set of subjects (n = 22).**

# References

Abe, T., Nonomura, T., Komada, Y., Asaoka, S., Sasai, T., Ueno, A., & Inoue, Y. (2011). Detecting deteriorated vigilance using percentage of eyelid closure time during behavioral maintenance of wakefulness tests. *International Journal of Psychophysiology*, *82*(3), 269–274. https://doi.org/10.1016/j.ijpsycho.2011.09.012

Caffier, P. P., Erdmann, U., & Ullsperger, P. (2003). Experimental evaluation of eye-blink parameters as a drowsiness measure. *European Journal of Applied Physiology*, *89*(3–4), 319–325. https://doi.org/10.1007/s00421-003-0807-5

Dinges, D. F., Mallis, M., Maislin, G., & Powell, J. W. (1998). *Evaluation of techniques for ocular measurement as an index of fatigue and the basis for alertness management.* (Report No. DOT HS 808 762). U.S. Department of Transportation, National Highway Traffic Safety Administration.

Roux, F. E., Djidjeli, I., & Durand, J. B. (2018). Functional architecture of the somatosensory homunculus detected by electrostimulation. *Journal of Physiology*, *596*(5), 941–956. https://doi.org/10.1113/JP275243

Yousry, T. A., Schmid, U. D., Alkadhi, H., Schmidt, D., Peraud, A., Buettner, A., & Winkler, P. (1997). Localization of the motor hand area to a knob on the precentral gyrus. A new landmark. *Brain*, *120 (Pt 1)*, 141–157. https://doi.org/https://doi.org/10.1093/brain/120.1.141
