# Supplementary material for: Somatotopic disruption of the functional connectivity of the primary sensorimotor cortex in complex regional pain syndrome type 1
Source: Hum Brain Mapp. 2023 Oct 14;44(17):6258–74. doi: 10.1002/hbm.26513 (PMC10619416; doi:10.1002/hbm.26513)
Supplement: Supplementary file 2 — FIGURE S1: Supplementary figures. [file HBM-44-6258-s001.docx]

# Supplementary Figure 1: Seed-to-seed functional connectivity analysis of the sensorimotor cortex


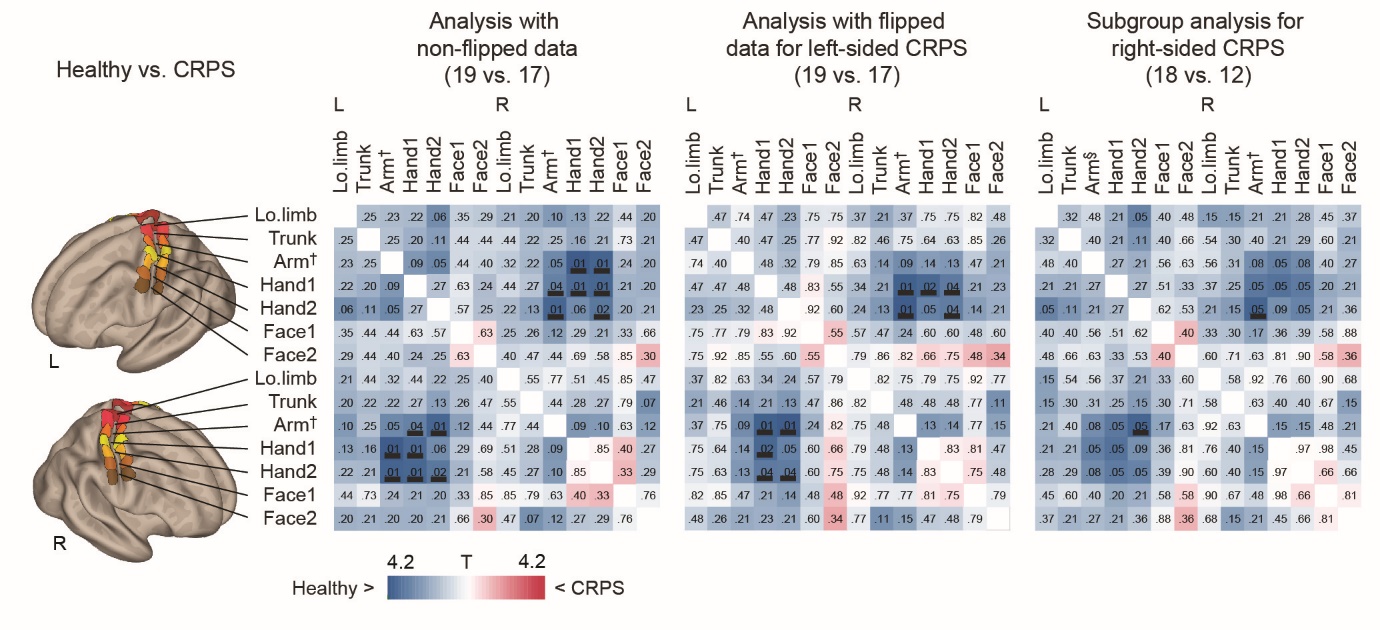


Supplementary Figure 1. Results of the seed-to-seed analyses of the 14 seeds at the sensorimotor cortex displayed separately for three different group comparisons: (1) using non-flipped data (left panel); (2) using flipped-data for left-pain patients and matched control subjects (middle panel), and on the right panel for the subgroup analysis of right-sided CRPS. The FDR-corrected p-values are displayed on top of the heat map that represents the t-values from the between-groups analyses. FDR-corrected p-values < 0.05 are underlined.

# Supplementary Figure 2: Group specific seed-to-voxel functional connectivity maps of the primary sensorimotor cortex upper-limb areas.

#
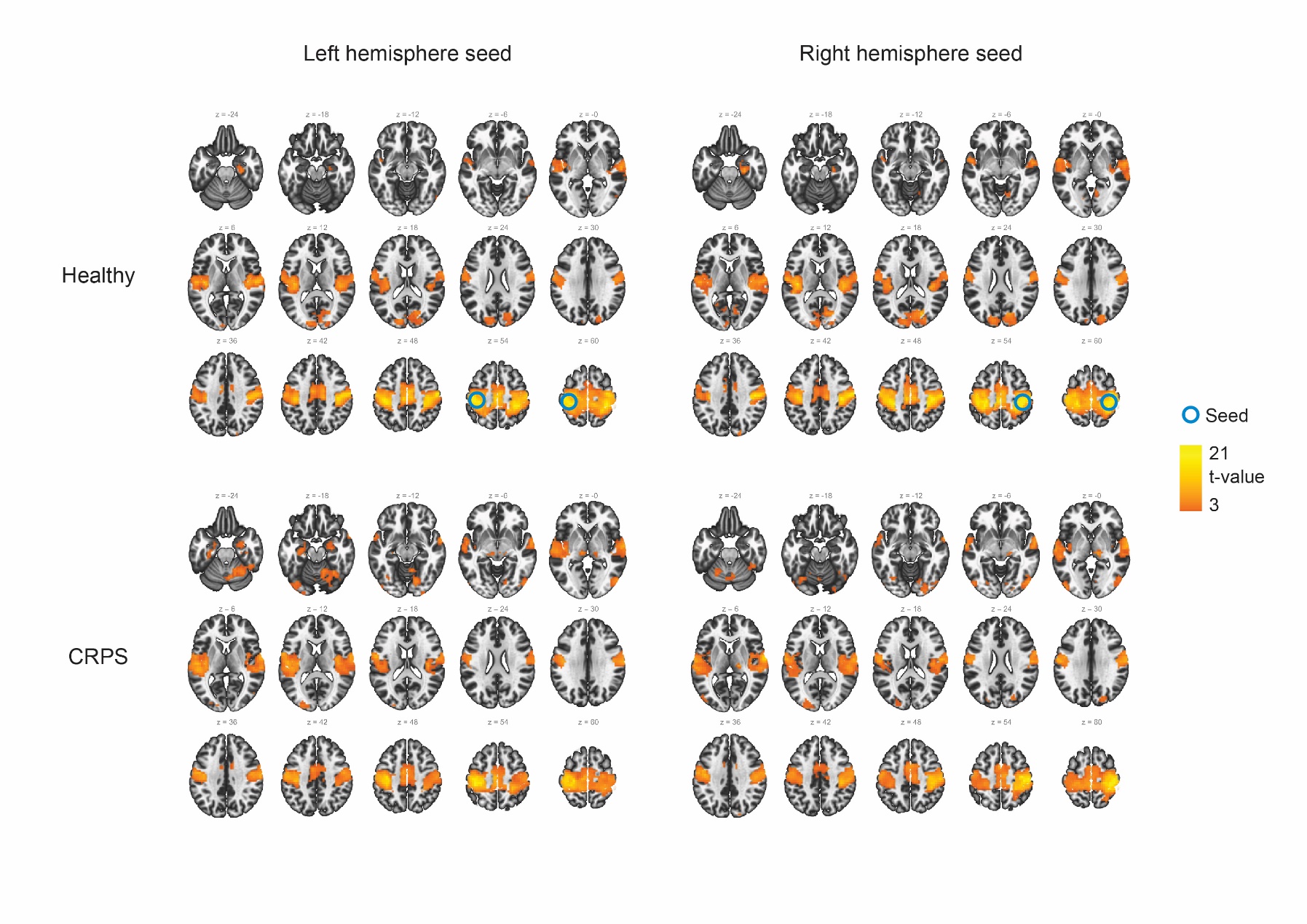


Supplementary Figure 2. The group specific seed-to-voxel functional connectivity maps displayed separately for seeds of the left and right sensorimotor cortex upper limb areas (UpperLimb_ROI_). For the visualization, the voxel-level statistical significance was set at p < 0.001 (uncorrected), together with cluster-level FDR corrected q < 0.05. To minimize between-group confounders, the group sizes were matched (n = 17 for both groups), together with best possible gender, age, and MRI-scanner match.

# Supplementary Figure 3: Seed-to-voxel functional connectivity analysis of the primary sensorimotor cortex upper-limb areas and independent component analysis of the sensorimotor network for the subgroup of right-sided CRPS


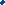

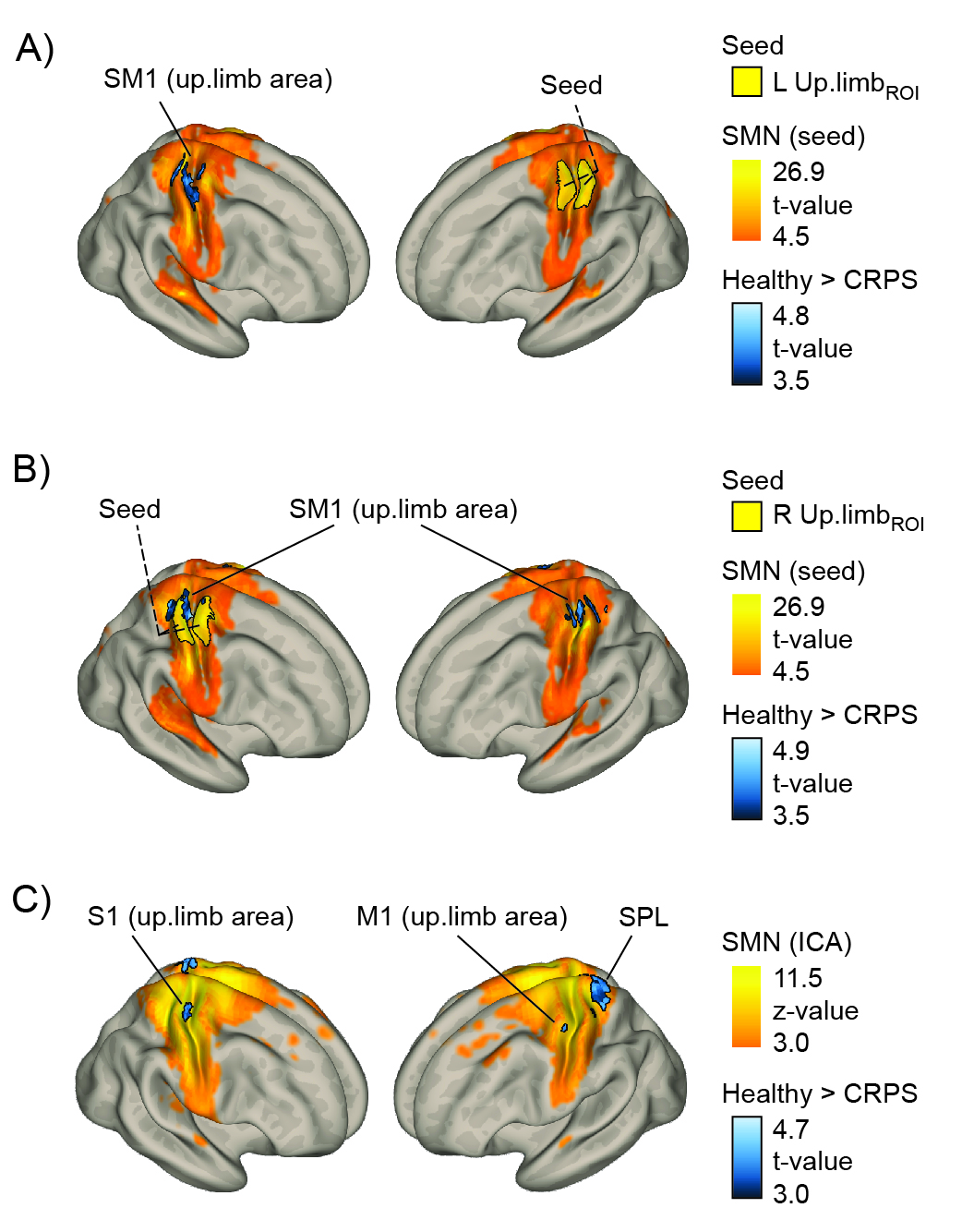


Supplementary Figure 3. (A) Seed-to-voxel subgroup analysis of patients suffering from right-sided CRPS with seeds in the left upper-limb sensorimotor cortex. The results are displayed on a 3D semi-inflated brain white-matter template (CONN toolbox, <https://www.nitrc.org/projects/conn>). The black-bordered black-to-blue gradient indicates statistically significantly decreased functional connectivity in the 12 CRPS patients compared with 18 healthy control subjects, and black-to-red gradient displays increased functional connectivity. The seeds are displayed in yellow (black-bordered). The sensorimotor network of the healthy control subjects was computed separately in each analysis and is displayed here with orange-to-yellow gradients. The thresholds for T and Z-values were chosen for visualization purposes; (B) Same as in (A) but for the seed in the right sensorimotor cortex; (C) Independent component analysis (ICA) of the sensorimotor network on a subgroup of 12 patients suffering from right-sided CRPS. M1 = primary motor cortex; PAG = periaqueductal gray matter; SM1 = primary sensorimotor cortex; SMN = sensorimotor network; SPL = superior parietal lobule; † the SM1 upper limb seed area, including the representation area of the upper limb from wrist to shoulder.

# Supplementary Figure 4: Post-hoc functional connectivity analysis for the periaqueductal gray matter


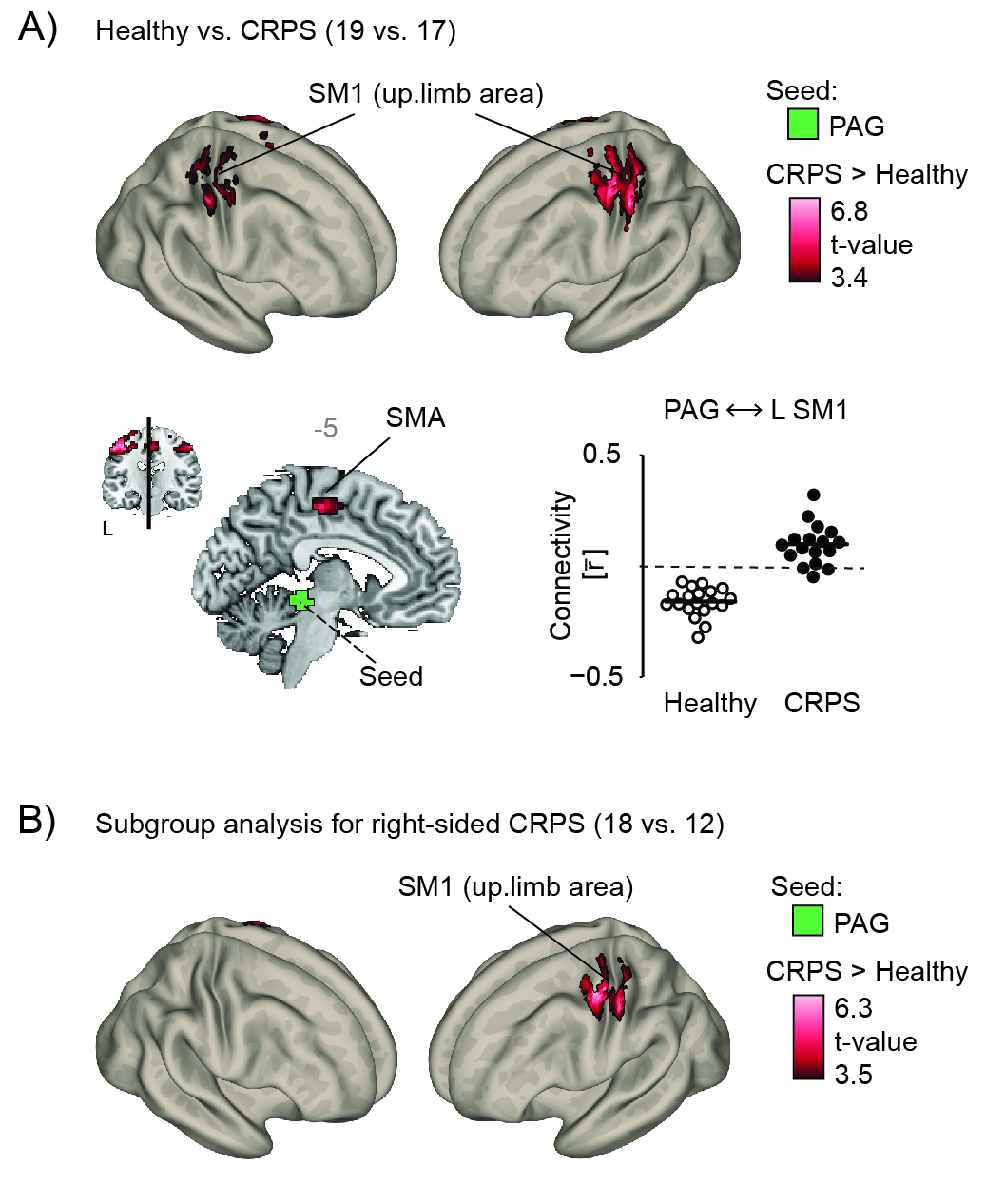


Supplementary Figure 4. Seed-to-voxel functional connectivity analysis with periaqueductal gray matter as the seed. (A) Comparison between healthy control subjects and CRPS patients (19 vs 17). The results are displayed on top of a 3D semi-inflated brain white-matter template (CONN toolbox, <https://www.nitrc.org/projects/conn>) and on representative slices of a template brain (MRIcroGL, <https://www.mccauslandcenter.sc.edu/mricrogl/home>). The black-bordered black-to-red gradient indicates statistically significantly increased functional connectivity in CRPS patients. The seed is displayed in green (black-bordered). (B) Same as in (A) but for the subgroup analysis including only right-sided CRPS (18 vs 12). PAG = periaqueductal gray matter; SM1 = primary sensorimotor cortex; SMA = supplementary motor area.

# Supplementary Figure 5: Seed-to-seed analysis of the functional connectivity between the sensorimotor cortex and right anterior insula: the additional analysis with flipped data for left-sided CRPS and the subgroup analysis for right-sided CRPS
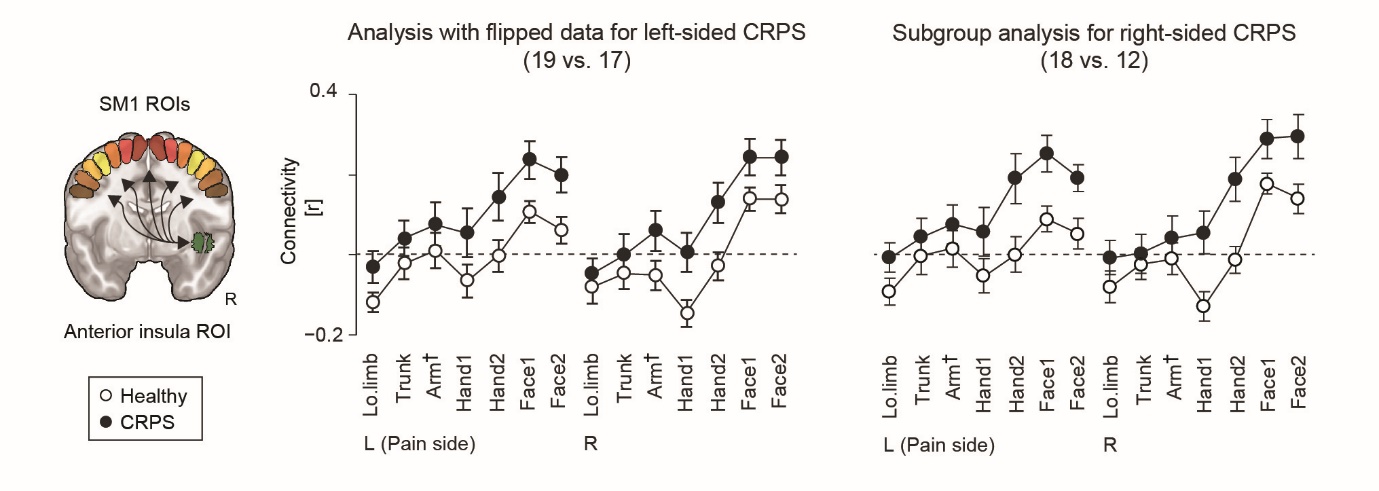


Supplementary Figure 5. The functional connectivity between the right anterior insula ROI and each of the fourteen ROIs of the sensorimotor cortex (seven on each hemisphere) plotted group-wise (mean ± SEM) for the analysis with flipped data of left-sided CRPS (left panel) and a subgroup analysis of right-sided CRPS (right panel). White-centred circles indicate the healthy control subjects and black circles the CRPS patients. All data corrected for the nuisance factors of age, MRI, PERCLOS and flipping of the data (if applied). L = left; R = right,; r = Pearson’s correlation; † = the ROI including representation area of the upper limb from wrist to shoulder.
