# Supplementary material for: Somatotopic disruption of the functional connectivity of the primary sensorimotor cortex in complex regional pain syndrome type 1
Source: Hum Brain Mapp. 2023 Oct 14;44(17):6258–74. doi: 10.1002/hbm.26513 (PMC10619416; doi:10.1002/hbm.26513)
Supplement: Supplementary file 3 — TABLE S1: Supplementary tables. [file HBM-44-6258-s003.docx]

Supplementary Table 1. Results of the between-group analysis for the nuisance factors. Drowsiness measured by percentage of eyelid closures.

|  | | | Patients | | Controls | | Mann-Whitney U-test  [p] |
| --- | --- | --- | --- | --- | --- | --- | --- |
|  |  |  | median | range | median | range |  |
|  | | |  |  |  |  |  |
| Percentage of eyelid closures [%] | | | 0.4 | 0.0–25.3 | 0.3 | 0.0–26.9 | 0.64 |
| Accelerometer magnitude vector | | |  |  |  |  |  |
|  | | |  |  |  |  |  |
|  | Mean power | |  |  |  |  |  |
|  |  | L | 46.1 | 21.6–456.6 | 65.9 | 21.3–1685.3 | 0.76 |
|  |  | R | 55.7 | 21.9–1084.3 | 53.5 | 19.3–1160.8 | 0.66 |
|  |  | \|L−R\| | 26.2 | 1.8–627.6 | 27.0 | 0.2–572.8 | 0.81 |
|  |  |  |  |  |  |  |  |
|  | L and R correlation [r] | | 0.16 | 0.02–0.38 | 0.16 | −0.06–0.61 | 0.89 |
|  | | | | | | | |

Supplementary Table 2. Results of the between-side analysis for the nuisance factors. Hand movements assessed by accelerometer measurements.

|  | | | Side 1 | | Side 2 | | Related Samples Wilcoxon Signed Rank-test  [p] |
| --- | --- | --- | --- | --- | --- | --- | --- |
|  |  |  | median | range | median | range |  |
| Accelerometer magnitude vector | | |  |  |  |  |  |
|  | Mean power | |  |  |  |  |  |
|  |  | L vs R (whole group, n = 34) | 55.8 | 21.3–1685.3 | 54.6 | 19.3–1160.8 | 0.94 |
|  |  | pain vs no-pain (patients, n = 15) | 45.8 | 21.6–456.60 | 55.7 | 21.9–1084.3 | 0.09 |
|  | | | | | | | |

Supplementary Table 3. Results of the seed-to-voxel subgroup functional connectivity analyses for 12 right-sided CRPS patients vs 18 healthy control subjects.

| Analysis | |  | Results | | | | | | | | | |
| --- | --- | --- | --- | --- | --- | --- | --- | --- | --- | --- | --- | --- |
|  |  |  | Cluster | | | | |  | Peak | | | |
|  |  |  |  |  |  | r* [mean ± SD] | |  |  |  |  | |
| Seed | Comparison |  | Area | Size [mm^3^] | *p*-FDR | CRPS | Healthy |  | x, y, z [MNI] | T | *p*-unc. | |
|  |  |  |  |  |  |  |  |  |  |  |  | |
| L Up.limb_ROI_ | Healthy > CRPS |  | R SM1 | 2953 | 0.001 | 0.23 ± 0.17 | 0.70 ± 0.13 |  | 34, −35, 59 | –4.8 | 0.000033 | |
|  |  |  |  |  |  |  |  |  |  |  |  | |
| R Up.limb_ROI_ | Healthy > CRPS |  | L SM1 | 2152 | 0.008 | 0.20 ± 0.12 | 0.63 ± 0.11 |  | −26, −31, 62 | –4.9 | 0.000024 | |
|  |  |  | R SM1 | 1477 | 0.021 | 0.42 ± 0.10 | 0.77 ± 0.09 |  | 26, −28, 53 | –4.8 | 0.000032 | |
|  |  |  |  |  |  |  |  |  |  |  |  | |
| PAG** | CRPS > Healthy |  | L SM1 | 6455 | 0.000001 | 0.19 ± 0.09 | −0.20 ± 0.11 |  | −41, −16, 56 | 6.3 | 0.000001 | |
|  |  |  |  |  |  |  |  |  |  |  |  | |
| * = corrected for nuisance factors of age, MRI and PERCLOS; ** = post-hoc analysis; L = left; PAG = periaqueductal gray matter; SM1 = primary sensorimotor cortex | | | | | | | | | | | |  |

Supplementary Table 4. Results of the sensorimotor network independent component analysis for 12 right-sided CRPS patients vs 18 healthy control subjects.

| Analysis |  | Results | | | | | |
| --- | --- | --- | --- | --- | --- | --- | --- |
|  |  | Cluster | |  | Peak | | |
| Comparison |  | Area | Size [mm^3^] |  | x, y, z [MNI] | T | *p*-TFCE |
|  |  |  |  |  |  |  |  |
| Healthy > CRPS |  | L SPL | 1392 |  | –23, –46, 59 | 4.3 | 0.023 |
|  |  | L M1 | 928 |  | –38, –16, 56 | 4.1 | 0.033 |
|  |  |  |  |  |  |  |  |
| Healthy > CRPS |  | L SPL | 2953 |  | –26, –43, 68 | 4.2 | 0.023 |
|  |  | R M1 | 380 |  | 26, –31, 53 | 4.7 | 0.025 |
|  |  | SMA | 84 |  | 0, –9, 59 | 3.5 | 0.047 |
|  |  | L M1 | 42 |  | –38, –16, 56 | 4.1 | 0.041 |
|  | | | | | | | |
